# ProNGF Drives Localized and Cell Selective Parvalbumin Interneuron and Perineuronal Net Depletion in the Dentate Gyrus of Transgenic Mice

**DOI:** 10.3389/fnmol.2017.00020

**Published:** 2017-02-09

**Authors:** Luisa Fasulo, Rossella Brandi, Ivan Arisi, Federico La Regina, Nicola Berretta, Simona Capsoni, Mara D'Onofrio, Antonino Cattaneo

**Affiliations:** ^1^Bio@SNS Laboratory, Scuola Normale SuperiorePisa, Italy; ^2^European Brain Research Institute Rita Levi-MontalciniRome, Italy; ^3^Department of Experimental Neurology, Fondazione Santa Lucia IRCCSRome, Italy

**Keywords:** proNGF, interneurons, parvalbumin, extracellular matrix, dentate gyrus, E/I imbalance, transgenic mice, expression profile

## Abstract

ProNGF, the precursor of mature Nerve Growth Factor (NGF), is the most abundant NGF form in the brain and increases markedly in the cortex in Alzheimer's Disease (AD), relative to mature NGF. A large body of evidence shows that the actions of ProNGF and mature NGF are often conflicting, depending on the receptors expressed in target cells. TgproNGF#3 mice, expressing furin-cleavage resistant proNGF in CNS neurons, directly reveal consequences of increased proNGF levels on brain homeostasis. Their phenotype clearly indicates that proNGF can be a driver of neurodegeneration, including severe learning and memory behavioral deficits, cholinergic deficits, and diffuse immunoreactivity for A-beta and A-beta-oligomers. In aged TgproNGF#3 mice spontaneous epileptic-like events are detected in entorhinal cortex-hippocampal slices, suggesting occurrence of excitatory/inhibitory (E/I) imbalance. In this paper, we investigate the molecular events linking increased proNGF levels to the epileptiform activity detected in hippocampal slices. The occurrence of spontaneous epileptiform discharges in the hippocampal network in TgproNGF#3 mice suggests an impaired inhibitory interneuron homeostasis. In the present study, we detect the onset of hippocampal epileptiform events at 1-month of age. Later, we observe a regional- and cellular-selective Parvalbumin interneuron and perineuronal net (PNN) depletion in the dentate gyrus (DG), but not in other hippocampal regions of TgproNGF#3 mice. These results demonstrate that, in the hippocampus, the DG is selectively vulnerable to altered proNGF/NGF signaling. Parvalbumin interneuron depletion is also observed in the amygdala, a region strongly connected to the hippocampus and likewise receiving cholinergic afferences. Transcriptome analysis of TgproNGF#3 hippocampus reveals a proNGF signature with broad down-regulation of transcription. The most affected mRNAs modulated at early times belong to synaptic transmission and plasticity and extracellular matrix (ECM) gene families. Moreover, alterations in the expression of selected BDNF splice variants were observed. Our results provide further mechanistic insights into the vicious negative cycle linking proNGF and neurodegeneration, confirming the regulation of E/I homeostasis as a crucial mediating mechanism.

## Introduction

ProNGF, the precursor of mature Nerve Growth Factor (NGF), is the most abundant NGF form in the brain and increases markedly in the cortex in Alzheimer's Disease (AD), relative to mature NGF (Francke et al., 1983; Scott et al., 1983; Fahnestock et al., 2001; Peng et al., 2004). Interestingly, signs of activated proNGF-signaling are detected both in AD and in preclinical mild cognitive impairment (MCI) (Mufson et al., 2012; Counts et al., 2016). A large body of evidence shows that the actions of ProNGF and mature NGF are often conflicting, depending on the receptors expressed in target cells: trkA, the preferred receptor for mature NGF; p75^NTR^, the pan-neurotrophin receptor, and, in association with its co-receptor sortilin (belonging to the VPS10 receptor family), a high affinity receptor for proNGF (Kaplan et al., 1991; Klein et al., 1991; Lee et al., 2001; Nykjaer et al., 2004; Masoudi et al., 2009). ProNGF can induce apoptosis in cells expressing p75^NTR^ and sortilin, regardless of the presence of trkA (Chao and Bothwell, 2002), and in different lesion models, generally characterized by a higher expression of p75^NTR^ (Beattie et al., 2002; Harrington et al., 2004).

In AD neurodegeneration, the activation of the amyloidogenic pathway has been demonstrated to promote proNGF/NGF dysmetabolism, shifting the balance of the processing reaction in favor of the precursor (Bruno et al., 2009; Iulita and Cuello, 2014).

Conversely, proNGF/NGF imbalance is itself a driver of neurodegeneration (Capsoni and Cattaneo, 2006), as shown, respectively, with indirect and direct evidence in AD11 (Ruberti et al., 2000) and TgproNGF#3 transgenic mice (Tiveron et al., 2013). AD11 mice, which express an antibody that neutralizes selectively mature NGF, with respect to proNGF, develop a progressive and comprehensive neurodegeneration (Capsoni et al., 2000) that can be fully rescued by NGF itself (De Rosa et al., 2005) or, in part, by p75^NTR^ gene ablation (Capsoni et al., 2010). TgproNGF#3 mice, expressing furin-cleavage resistant proNGF in CNS neurons, directly reveal consequences of increased proNGF levels on brain homeostasis. Their phenotype clearly indicates that proNGF can be a driver of neurodegeneration, including severe learning and memory behavioral deficits, cholinergic deficits, and diffuse immunoreactivity for A-beta and A-beta-oligomers (Tiveron et al., 2013). Interestingly, in aged TgproNGF#3 mice spontaneous epileptic-like events are detected in entorhinal cortex-hippocampal slices, suggesting the occurrence of excitatory/inhibitory (E/I) imbalance, whereas *in vivo* no spontaneous seizures were observed (Tiveron et al., 2013).

In such a view, the molecular events linking increased proNGF levels to the observed *in vitro* epileptiform activity in hippocampal slices deserve further investigation.

The occurrence of spontaneous epileptiform discharges in the hippocampal network in TgproNGF#3 mice suggests an impaired inhibitory interneuron homeostasis.

NGF/proNGF balance was demonstrated to deeply affect cholinergic phenotype (Capsoni and Cattaneo, 2006; Capsoni et al., 2010). Pharmacologically-induced chronic failure in extracellular NGF maturation leads to a reduction in mNGF levels, proNGF accumulation, cholinergic degeneration, and cognitive impairment in rats (Allard et al., 2012). TgproNGF#3 mice directly reveal the impact of increased proNGF levels and NGF/proNGF imbalance on the cholinergic phenotype, presenting a marked cholinergic deficit in BFCN projecting to cortex, and hippocampus in 3-month-old mice (Tiveron et al., 2013). No deficit is observed earlier, at 1-month.

Cholinergic modulatory activity has been demonstrated to affect hippocampal Parvalbumin interneurons subpopulation essential in determining the oscillatory activity (Gulyás et al., 2010; Lawrence et al., 2015). We thus propose that pathological conditions characterized by increased levels of proNGF in the brain, might lead to a reduced cholinergic drive to interneurons, with the ensuing E/I imbalance. In the present study we therefore evaluated the interneuron subpopulations in the hippocampus of TgproNGF#3 mice, at different ages. Parvalbumin+ (Parv+) interneurons are ensheathed by an aggregation of proteoglycan extracellular matrix (ECM) components, the perineuronal net (PNN), that reaches full maturation only during post-natal development and plays an essential role in regulating neuronal firing, stabilizing synapses and regulating synaptic plasticity (Pizzorusso et al., 2002; Wang and Fawcett, 2012). Impairment in PNN structures is described in condition of enhanced neuronal activity (McRae et al., 2012). We therefore also analyzed the PNN system. The robust transcriptional effects of NGF are well-characterized (Dijkmans et al., 2009). More recent data show that proNGF activates a largely distinct transcriptional program, and is a less potent transcriptional activator, compared to NGF, in target cells *in vitro* (D'Onofrio et al., 2011); moreover the extent of NGF/proNGF imbalance, affects the transcriptional outcome (Arisi et al., 2014). Therefore, a parallel analysis of early transcriptional changes in transgenic mice hippocampus was performed, in order to gain further insights into the mechanism starting and sustaining TgproNGF#3 mice phenotype.

## Materials and methods

### Animal handling and experiments

All experiments with mice were performed according to the national and international laws for laboratory animal welfare and experimentation (EEC council directive 86/609, 12 December 1987, and Leg. Decree n°26, Implementation of the UE Directive 2010/63/UE, 4 March 2014). The experiments were performed according to a protocol approved by the Italian Ministry of Health with the authorization n. 3/2012 issued on February 21st 2012, valid until February 21st 2015, in accordance with the guidelines and regulations of the Italian Law (DLGs n.116, 27/1/1992), and carried out in that time interval. Mice were kept under a 12 h dark to light cycle, with food and water *ad libitum*. Genotyping was performed as described in Tiveron et al. (2013).

#### Transgenic mice

As we previously reported in Tiveron et al. (2013), the mouse pre-proNGF cDNA was mutagenized at the furin cleavage site (Beattie et al., 2002) and placed under the transcriptional control of the mouse Thy1.2 promoter, driving brain-specific expression in post-natal and adult transgenic mice (Tiveron et al., 2013). The furin-resistant pre-proNGF cDNA, plus IRES sequence and EGFP, were cloned into the Thy1.2 promoter vector (Caroni, 1997), containing 6.5 kb of the murine thy1.2 gene, driving brain-specific expression in adult transgenic mice. The fragment includes exons I, II, and IV of the mouse Thy 1.2 gene (Vidal et al., 1990), responsible for the selective expression in neuronal cells, whereas exons containing the Thy 1.2 coding region or those responsible for expression in the thymus are absent. Transgenic mice were generated by pronuclear DNA injection of zygotes C57Bl/6xDBA/2 F2 generation using standard procedures (Ciana et al., 1998). Microinjected zygotes were reimplanted into pseudopregnant C57Bl/6xDBA/2 F1 foster mothers to complete their development. For genotyping genomic DNA was extracted from tail biopsies.

#### Primers for genotyping

IRES-EGFP 4F: (s) gga cgt ggt ttt cct ttg aa;

IRES-EGFP 5R: (as) gtc ctc ctt gaa gtc gat gc.

#### Histological analysis: immunofluorescence and image analysis

ProNGF and WT mice were anesthetized with an excess of 2,2,2-tribromethanol (400 mg/kg) and intracardially perfused with a saline solution and following a 4% solution of paraformaldehyde in phosphate buffer saline (PBS). Brains were post-fixed for 24 h then transferred in 30% sucrose/PBS solution and sectioned on a sliding freezing microtome (Leica, Wetzlar, Germany). Forty micrometers coronal sections were collected in 0.05% sodium azide/PBS in 1.5 ml tubes and stored at 4°C until usage. Immunofluorescence (IF) stainings were performed using the following primary antibodies: rabbit anti-proNGF antibody (dilution 1:100, Millipore Merkgroup, Italy), rabbit anti-aggrecan antibody (1:100 dilution, Millipore, Merkgroup, Italy), mouse anti-Parvalbumin antibody (1:800 dilution, Millipore Merkgroup, Italy) rabbit anti-GAD65-67 antibody (1:1000 dilution, Millipore Merkgroup, Italy), rabbit anti-calbindin 28k antibody (1:1000 dilution, Swant, Switzerland), PNN was stained using biotinylated lectin from Wisteria Floribunda (1:800 dilution, SIGMA, Italia), followed by FITC-, Texas Red-, or AMCA-Streptavidin (Vector Labs, UK).

Double-IF was performed using Alexa 594 and Alexa 488 secondary antibodies (1:200, Invitrogen). Immunofluorescence was examined under a confocal laser-scanning microscope (Leica SP5, Leica Microsystems, Wetzlar, Germany). Confocal acquisition settings were consistent between wild type and transgenic cases. Negative controls for secondary antibodies are shown in Figure S1.

Images were acquired with 5X and 20X dry objective and 40/63X oil immersion objectives at 1024 × 1024 pixel resolution. Settings for laser intensity, gain, offset, and pinhole were optimized initially and held constant through the experiment.

Final figures were assembled using Adobe Photoshop 7 and Adobe Illustrator 10 and Omnigraffle Professional. Image analysis was performed under visual control to determine thresholds that subtracts background noise and take into account neuronal structures with ImageJ software (US NIH) or Imaris Suite 7.4® (Bitplane A.G., Zurich, Switzerland). During image processing, the images were compared with the original raw data to make sure that no structures were introduced that were not seen in the original data series or that structures present in the original data series were not removed.

Cell counting was performed for each region on two squares of 450 × 450 μm per brain section, at least 4–6 sections per animal, females, and males (*n* = 4 per each animal group). Cell counting was performed on the hippocampus and DG subregion on both side or on the lateral region of the amygdala on both sides. Cells positive for each marker or double stained were counted manually and their density was calculated. Stereology counting was applied (Capsoni et al., 2010). Cell counting was expressed as cell density (Mainardi et al., 2009; Cardoso et al., 2013) per volume (cells/mm3). All analyses were done using a blind procedure. Statistical analysis: all data are expressed as mean ± *S.E.M*. Statistical analyses were performed using Student's *t*-test (two-tailed distribution) Differences were considered significant at *p* < 0.05.

#### Microarray analysis: rna isolation, hybridization, and analysis

Hippocampus (HP) of the right hemisphere was dissected from the brains of freshly sacrificed mice. All the tissue samples were stored in RNAlater (QIAGEN, UK). Total RNA was isolated from this brain area, using Trizol (Invitrogen S.R.L., Life Technologies, Italy) and DNAse, by Qiagen columns. RNA quantity was determined on a NanoDrop UV-VIS. Only samples with an absorbance ratio in the range 1.8 < OD260/OD280 < 2.0 were selected. Quality of RNA samples was checked for integrity with the Agilent BioAnalyzer 2100 (Agilent RNA 6000 nano kit, Agilent Technologies, Inc., Santa Clara, CA, USA): samples with a RIN index lower than 8.0 were discarded.

The gene expression profiling was performed using the One-Color Microarray Agilent Platform according to the Agilent protocol (Agilent 8X60K whole mouse genome oligonucleotide microarrays, GRID ID 028005). Data extraction from the Agilent scanner images was accomplished by Feature Extraction software. Data filtering and analysis were performed using Agilent GeneSpring GX 11.0, Microsoft Excel and R-Bioconductor (Limma package). All the features too close to background (with the flag gIsWellAboveBG = 0 in raw data files generated by Feature Extraction software) were filtered out and excluded from the following analysis. Filtered data were normalized by aligning samples to the 75th percentile in Log2 scale. Differentially expressed genes were selected by a combination of fold change and moderated *T*-test thresholds (R Limma-test *p* < 0.05; fold change ratio transgenic/WT >2.0 OR < 0.5 in linear scale). The analysis of over- and under-represented functional gene categories was performed using the DAVID web tool (https://david.ncifcrf.gov) and Gene Set Enrichment Analysis (GSEA) (http://software.broadinstitute.org/gsea/index.jsp) (Subramanian et al., 2005; Huang et al., 2009).

The following link provides information about how to interpret GSEA data http://software.broadinstitute.org/gsea/doc/GSEAUserGuideFrame.html?Interpreting_GSEA. All gene expression microarray data are publicly available in the Gene Expression Omnibus database at the following link: https://www.ncbi.nlm.nih.gov/geo/query/acc.cgi?acc=GSE70757.

### Real-time qRT-PCR

RNA was isolated, quality controlled as described above, and subjected to quantitative real-time RT-PCR (qRT-PCR) using the two-step iCycler iQ5 Real-Time Detection system (Bio-Rad, USA). For quantification of gene expression changes, the ΔΔCt method was used to calculate relative fold changes normalized against the housekeeping gene Peptidylprolyl Isomerase A (Ppia). Each data point was obtained from four biological replicates (four mice TgproNGF#3 and four age-matched control mice), each of them in duplicate. Error bars were computed according to the standard error of the mean and the error propagation. The one-tail *T*-test, assuming equal variances, was used to select significant expression values.

Primers for real time (qRT-PCR) analysis were selected from Harvard Primer database and are listed below.

#### Primers for real-time qRT-PCR analysis

**Kcc2** (**Slc12a5**) (**s**)gggcagagagtacgatggc; (**as**)tggggtaggttggtgtagttg;
**Nkcc1** (**Slc12a2**) (**s**) ttccgcgtgaacttcgtgg; (**as**) ttggtgtgggtgtcatagtagt;
**Calm3** (**s**) tctccctcttcgacaaggatg; (**as**) ggttctgtcccagcgatctc;
**Camk2a**(**s**) tggagactttgagtcctacacg; (**as**) ccgggaccacaggttttca;
**Dlg4**(**s**) tccgggaggtgacccattc; (**as**) tttccggcgcatgacgtag;
**Eif2s1**(**s**) atgccggggctaagttgtaga; (**as**) aacggatacgtcgtctggata;
**H3f3a** (**s**) tgtggccctccgtgaaatc; (**as**) ggcataattgttacacgtttggc;
**Hist2h4** (**s**) ggtggaaagggtctaggcaag; (**as**) cctggatgttgtcacgcaaga;
**Aph1b**(**s**) tcactggaatcagttggctct;(**as**) catccgggaagatgatcagta;
**Slc6a13**(**s**) acctgtgagcctggctgt; (**as**) ccaccacagaggggtagttc;
**Lama1**(**s**) cagcgccaatgctacctgt; (**as**)ggattcgtactgttaccgtcaca;
**Col6a2**(**s**) gatctgttagaccgccatgc; (**as**) cagggctagggtcctattagc;
**Col3a1**(**s**) ctgtaacatggaaactggggaaa; (**as**) ccatagctgaactgaaaaccacc.

#### Specific primers for BDNF

**BDNF1** (**s**)agtctccaggacagcaaagc (**as**)tgcaaccgaagtatgaaataacc
**BDNF2A (s)**gatcccggagagcagagtc**(as)**tctcacctggtggaactgg
**BDNF2B (s)**gcggtgtaggctggaataga**(as)**aaggatggtcatcactcttctca
**BDNF2C (s)**gtggtgtaagccgcaaaga**(as)**aaccatagtaaggaaaaggatggtc
**BDNF3 (s)**gagactgcgctccactcc**(as)**aaggatggtcatcactcttctca
**BDNF4 (s)**gctgccttgatgtttactttga**(as)**aaggatggtcatcactcttctca
**BDNF5 (s)**gatccgagagctttgtgtgg**(as)**aaggatggtcatcactcttctca
**BDNF6A (s)**ctcctgaggaagtgaaagttttg**(as)**aaggatggtcatcactcttctca
**BDNF6B (s)**ccgaacaaactgattgctga**(as)**aaggatggtcatcactcttctca
**Exon 8** (shared between long and short BDNF mRNAs)
**(s)**gcctttggagcctcctctac**(as)**gcggcatccaggtaatttt
**Exon 8 IIPolyA**(upstream the second polyA, amplifies long BDNF mRNAs)
**(s)**gctctcttacccactaagatacatca **(as)**ttttaacaaataaatctcaggtcaaca
(according to Liu et al., 2006; Juan et al., 2008).

### Electrophysiology

For multisite recordings, combined entorhinal cortex-hippocampal (EC-hippocampal) slices (350 μm) were cut according to the method described by Jones and Heinemann (1988) and then incubated in ACSF. To evaluate epileptiform activity, EC-hippocampal slices were placed over an 8 × 8 multi-electrode array (MEA) of planar electrodes, each 50 × 50 μm in size, with an interpolar distance of 300 μm (MED-P5305; Alpha MED Sciences, Kadoma, Japan) under visual control, so that signals from each electrode could be assigned according to their relative position within the hippocampal-entorhinal area (Berretta et al., 2012; Tiveron et al., 2013). Slices were kept submerged in ACSF with a nylon mesh glued to a platinum ring and continually perfused in ACSF (6 ml/min) at 34°C. Voltage signals were acquired using the MED64 System (Alpha MED Sciences, Kadoma, Japan), digitized at 20 kHz and filtered (0.1–1 Hz) with a 6071E Data Acquisition Card (National Instruments, Austin, USA), using Mobius software (Alpha MED Sciences, Kadoma, Japan).

## Results

### Parvalbumin interneurons are selectively reduced in the dentate gyrus (DG) and amygdala of TgproNGF#3 mice

The occurrence of spontaneous epileptiform discharges in the hippocampal network in aged TgproNGF#3 mice (Tiveron et al., 2013) suggests impaired inhibitory interneuron homeostasis. We therefore evaluated different interneuron inhibitory subpopulations in TgproNGF#3 mice hippocampus. A pilot survey in the hippocampus demonstrated that while no major changes were observed in the number of calretinin and calbindin interneurons (Figures S2, S3), the number of Parv+ interneurons appeared to be strikingly affected in the DG.

**Figure 1:**
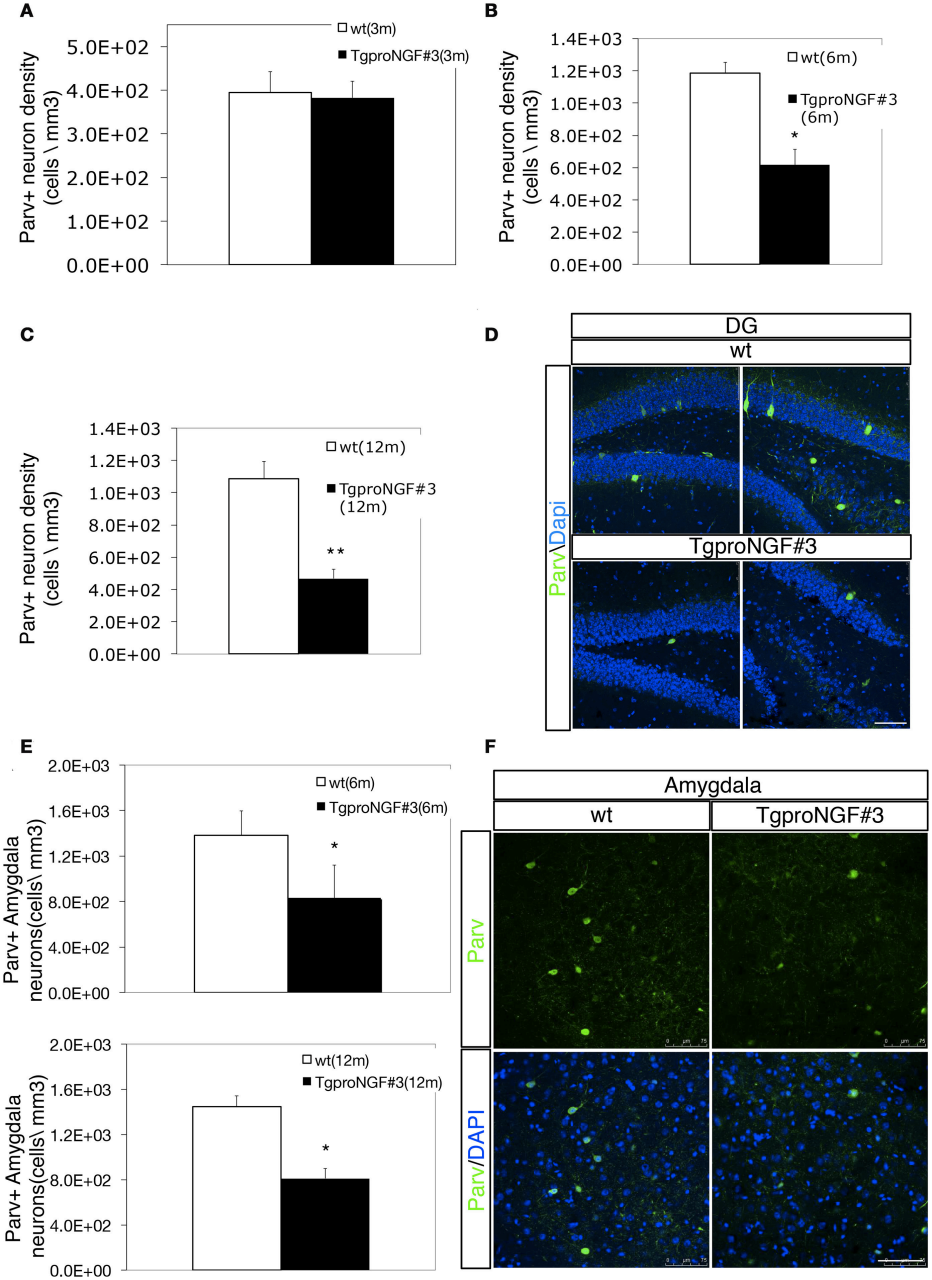
**(A–D)** Parvalbumin+ interneuron depletion in the DG of TgproNGF#3 mice. **(A–C)** Quantification of Parvalbumin+ interneurons (Parv+ interneuron density, expressed as cells/mm^3^) in the DG of wt and TgproNGF#3 mice (3, 6, and 12 ms-old). Cell counting was performed on at least 4–6 sections per animal, males and females (*n* = 4 per each animal group). Bars and lines are representative of mean ± SEM (*T*-test, **P < 0.005; *P < 0.05). **(D)** Confocal micrographs of Parvalbumin immunofluorescence in 12-month-old wt and TgproNGF#3 mice hippocampus (DG region). Scale bar: 75 micron. **(E,F)** Parvalbumin+ interneuron depletion in the amygdala of 12-month-old TgproNGF#3 mice. **(E)** Quantification of Parv+ interneurons in the basolateral amygdala of 6 and 12-month-old wt and TgproNGF#3 mice (Parv+ interneuron density, expressed as cells/mm^3^). Cell counting was performed on the lateral region of the amygdala on both sides on at least 4–6 sections per animal, males and females (*n* = 4 per each animal group) (*T*-test, *P < 0.05). **(F)** Confocal micrographs of Parvalbumin immunofluorescence labeling in the amygdala of 12-month-old wt and TgproNGF#3 mice (Parv+ neurons are located in the lateral region of the amygdala). Scale bar: 75 micron.

In wt with the same genetic background of TgproNGF#3 mice, the Parv+ neuron density in the DG (Figures 1A–D) was in accordance to the literature (Takahashi et al., 2010; Cardoso et al., 2013). In 3-month-old TgproNGF#3 mice hippocampus, no reduction in the total number of Parv+ cells in the DG was observed (Figure 1A). Later, in 6- and 12-months-old TgproNGF#3 mice, the number of Parv+ interneurons was markedly reduced in the DG (47 and 53%, respectively, of the number in age-matched controls (Figures 1B–D). Of note, the number of Parv+ neuron was not affected in other hippocampal regions, such as CA1 (Figure S4), of TgproNGF#3 mice, showing that the effect is regional-selective.

Since Active Avoidance behavioral response is severely impaired in TgproNGF#3 mice (Tiveron et al., 2013), the Parvalbuminergic interneuron subpopulation was evaluated also in the basolateral amygdala, a region involved in avoidance responses (Choi et al., 2010; Tiveron et al., 2013) and strongly connected to the hippocampus. Interestingly, also in the lateral amygdala a marked reduction of Parv+ interneurons was observed in 6–12 months TgproNGF#3 mice (40 and 44%, respectively, of the number of age-matched control Figures 1E,F), whereas no change was observed at 3-months (not shown).

**Figure 2:**
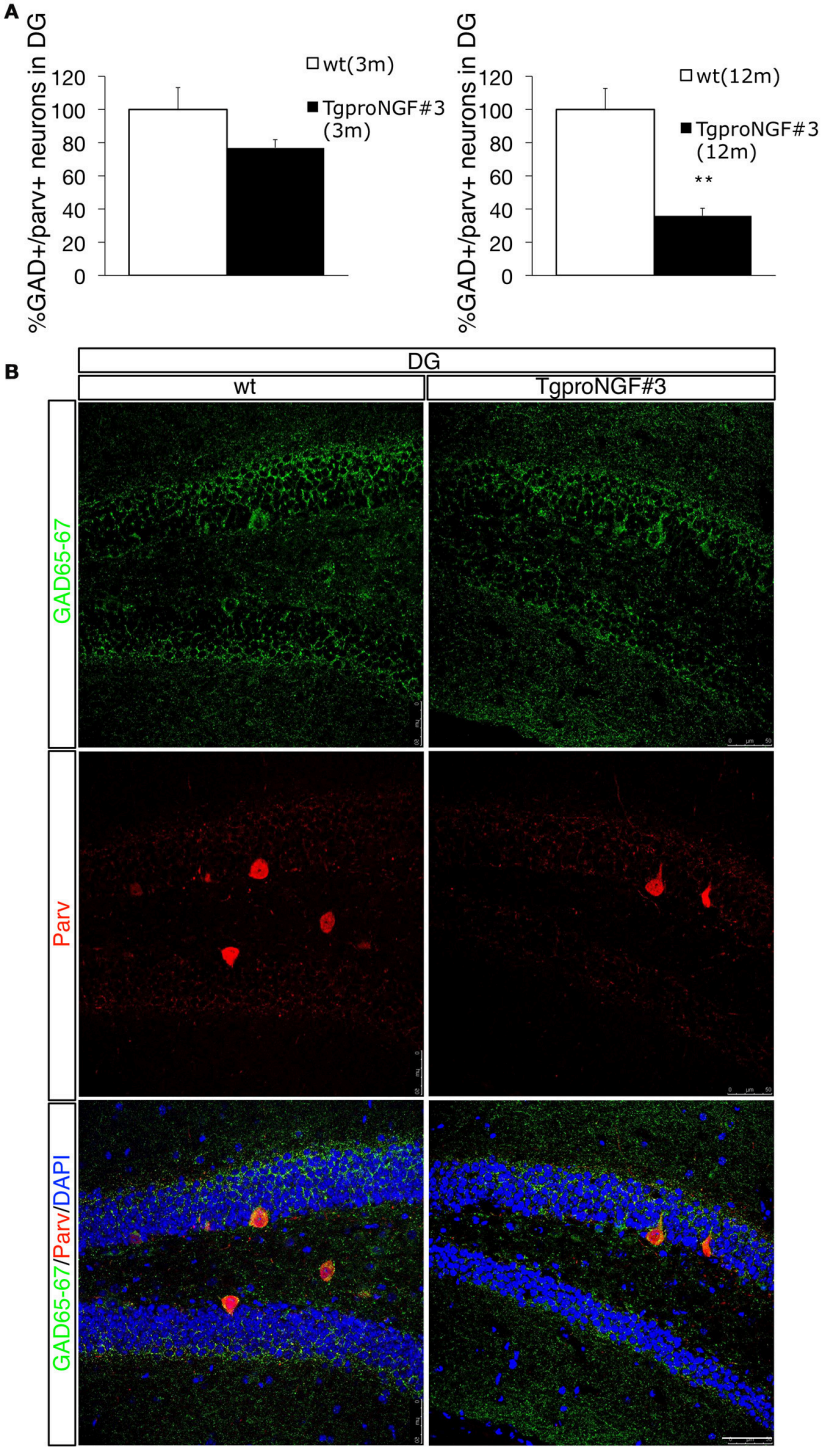
**Double labeling for Parvalbumin and GAD65-67 in the dentate gyrus (DG) of 12-months-old wt and TgproNGF#3 mice. (A)** Quantification of GAD+/Parv+ neurons of wt and TgproNGF#3 mice (3- and 12-month old), expressed as percentage. Cell counting was performed on the DG on both sides, on at least 4–6 sections per animal, males and females (*n* = 4 per each animal group) (*T*-test, **P < 0.005). **(B)** Confocal Micrographs of Parv/GAD65-67 double immunofluorescence labeling in the DG of wt and TgproNGF#3 mice. Reduction of both Parv and GAD markers was observed. Scale bar: 50 micron.

Double labeling with antibodies to Parvalbumin and GAD65-67 (a marker for GABAergic interneurons that does not strongly label DG principal granule cells, Figure 2B, upper panels) showed, in the DG of 12-month-old TgproNGF#3 mice, a reduction in the number of Parv/GAD65-67 double-labeled neurons (Figures 2A,B), a sign of neuron loss at this age or of reduced GAD protein expression. However, the change was selectively restricted to the DG, therefore biochemically undetectable. No significant decrease of Parv/GAD65-67 double-positive interneurons was observed, instead, at 3-months (Figure 2A).

However, selective vulnerability of Parv+ interneurons in the DG cannot be ascribed to a higher level of proNGF expression in this hippocampal subregion, as revealed by the very low proNGF immunoreactivity (Figure S5). Determination of transgenic furin-resistant proNGF transcript (vs. the endogenous one) and proNGF/NGF protein levels in the hippocampus and other brain regions were previously reported (Tiveron et al., 2013).

TgproNGF#3 mice show therefore an impaired inhibitory interneuron homeostasis, as revealed by a regional- and cell type-selective Parv interneuron depletion in the DG that might be the cause of the epileptiform discharges observed in the hippocampal network.

### CB immunoreactivity is selectively reduced in DG granule cells and mossy fibers of aged TgproNGF#3 mice

**Figure 3:**
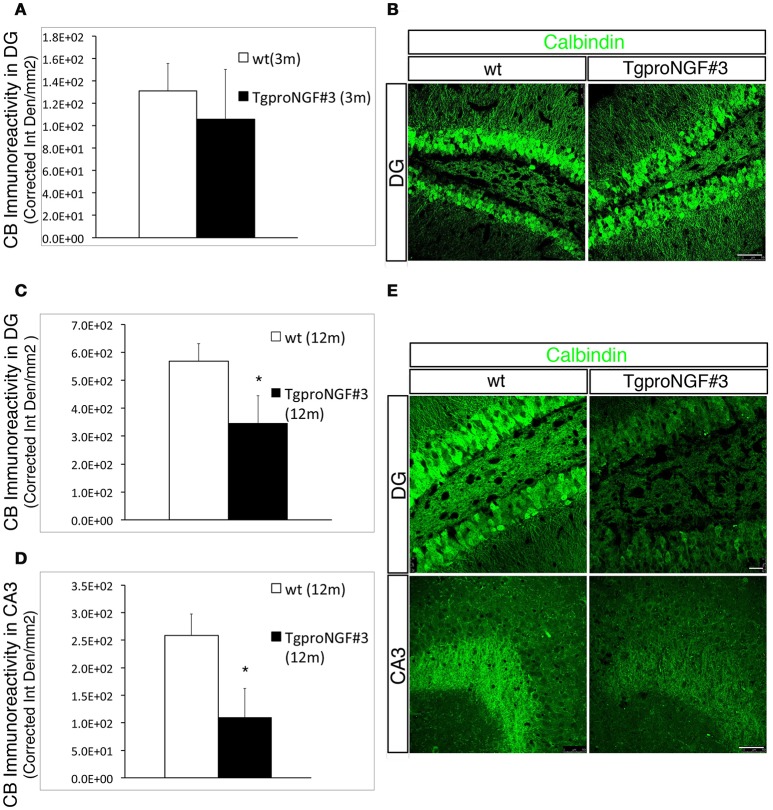
**Calbindin immunoreactivity is unaffected in 3-month-old TgproNGF#3 mice, but markedly reduced in the DG and CA3 region of the hippocampus of 12-month-old TgproNGF#3 mice. (A)** Quantification of fluorescence intensity for calbindin (expressed as Corrected Integrated Density/mm^2^) in the DG region of 3-month-old wt and TgproNGF#3 mice. **(B)** Confocal micrographs of calbindin immunofluorescence labeling in 3-month-old wt and TgproNGF#3 mice (DG region of the hippocampus). Scale bar: 50 micron. **(C)** Quantification of fluorescence intensity for calbindin (expressed as Corrected Integrated Density/mm^2^) in the DG region of 12-month-old wt and TgproNGF#3 mice (*T*-test, *P = 0.01). **(D)** Quantification of fluorescence intensity for calbindin (expressed as Corrected Integrated Density/mm^2^) in the CA3 region of 12-month-old wt and TgproNGF#3 mice (*T*-test, *P = 0.01). Analysis was performed on at least 4–6 sections per animal, males and females (*n* = 4 per each animal group). **(E)** Reduced Calbindin immunostaining in DG region of the hippocampus (upper panels) and in CB+ axons (Mossy fibers) projecting to the CA3 region (lower panels) in 12-month-old wt and TgproNGF#3 mice. (**E**, upper panels) Confocal micrographs of calbindin immunofluorescence labeling in 12-month-old wt and TgproNGF#3 mice in DG region of the hippocampus. Scale bar: 25 micron. (**E**, lower panels) Confocal micrographs of calbindin immunofluorescence labeling in 12-month-old wt and TgproNGF#3 mice in CA3 region in 12-month-old wt and TgproNGF#3 mice. Scale bar: 50 micron.

Calbindin (CB) is another cytosolic Ca^++^ buffer protein expressed in inhibitory interneurons and in granule cells. We evaluated whether the CB+ interneurons and /or the CB levels were also reduced in the hippocampus of TgproNGF#3 mice. No significant change was observed in the density of CB-positive interneurons in the hippocampus of TgproNGF#3 mice, as revealed by hippocampal calbindin immunoreactivity (Figure S3). Moreover, no significant reduction of CB-immunoreactivity was observed in granule cells in the DG in 3-month-old TgproNGF#3 mice (Figure 3B, quantification in **A**). A striking depletion was instead observed in granule cells in the DG of 12-month-old TgproNGF#3 mice (Figure 3E, upper panels, quantification in **C**). In the same region, granule neurons number (Figure S8) as well as the GCL area were unaffected, thus demonstrating that the reduced CB-immunoreactivity indicates depletion of CB protein, also observed in CB+ mossy fiber axons projecting to CA3 (Figure 3E, lower panels, quantification in **D**). The reduction of CB-immunoreactivity was region-specific, as it was not observed in other hippocampal regions.

### Perineuronal nets are progressively and selectively depleted in the DG of TgproNGF#3 mice

**Figure 4:**
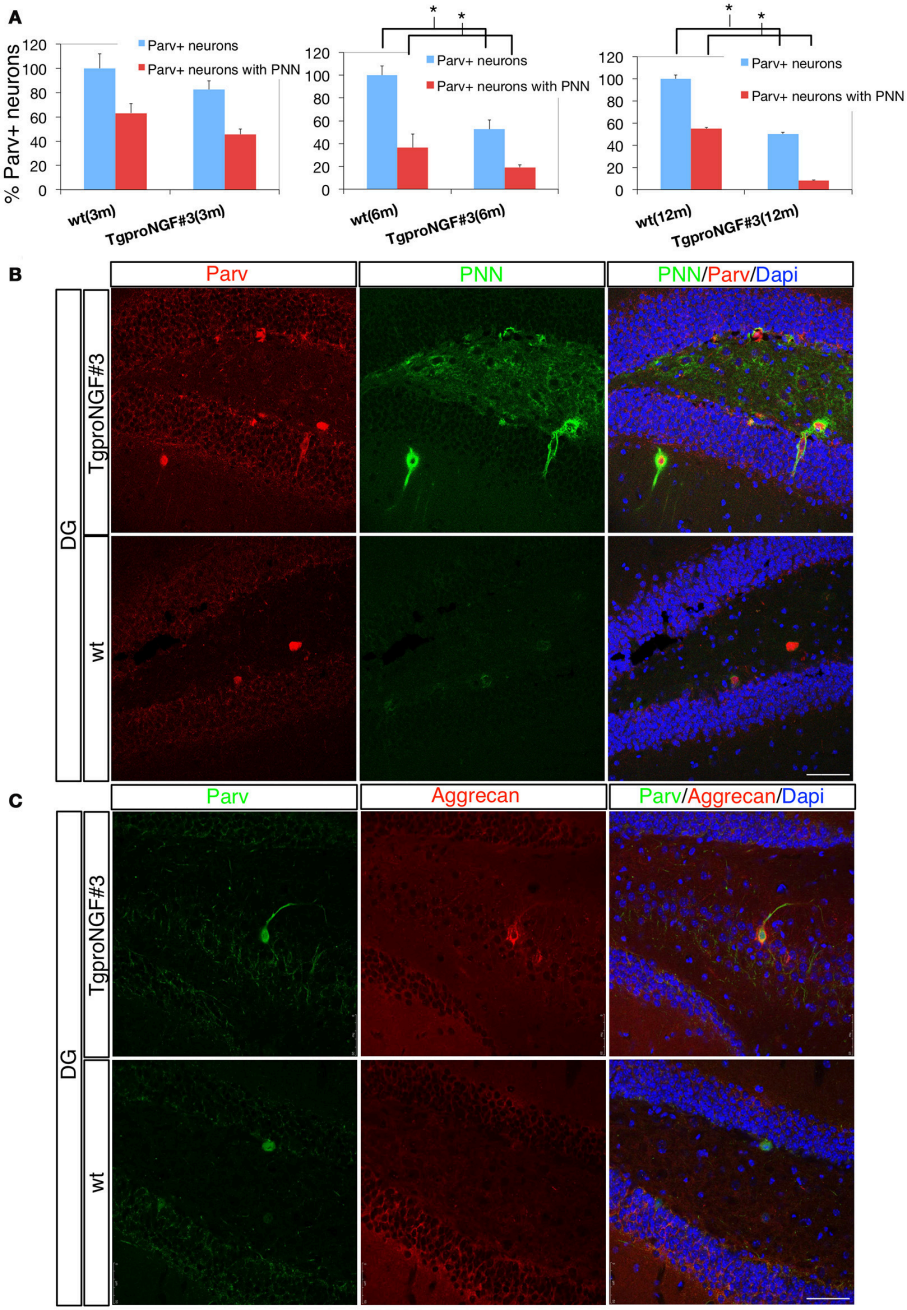
**(A,B)** The perineuronal nets (PNN) are markedly reduced in the dentate gyrus of TgproNGF#3 mice. Confocal microscopy. **(A)** Quantification of Parvalbumin+ interneurons of wt and TgproNGF#3 mice (3-, 6-, and 12-month-old). % total Parv+ neurons − *blue bar* − and Parv+ neurons ensheathed by the PNN − *red bar*. The reduction in Parv+ neurons ensheathed by PNN is significant in 6- and 12-month TgproNGF#3 mice. Analysis was performed on at least 4–6 sections per animal, males and females (*n* = 4 per each animal group) (*T*-test, *P < 0.005). **(B)** Double immunofluorescence for the perineuronal net and for Parvalbumin in the DG of 12-months-old wt and TgproNGF#3 mice. Brain sections were labeled with anti-Parvalbumin antibody, with the lectin Wisteria Floribunda (an agglutinin commonly used to map PNN and ECM, since it binds the glycan component of PNN proteoglycans) and Dapi. Scale bar: 50 micron. **(C)** Double immunofluorescence for aggrecan (a component of the PNN surrounding Parv+ interneurons) and for Parvalbumin in the DG of 12-months old wt and TgproNGF#3 mice. Scale bar: 50 micron.

Since Parv+ interneurons are ensheathed by the PNN, playing an essential role in synaptic maturation, we evaluated PNN in the DG of TgproNGF#3 mice (Figures 4A,B). The lectin from Wisteria Floribunda (an agglutinin commonly used to map PNN and ECM) only faintly decorates the DG in 12-month-old TgproNGF#3 mice, compared to the much stronger labeling of WT mice (Figure 4B), providing evidence for a broad down-regulation of ECM protein components, specifically at these important extracellular structures. The deficit appeared to be specific for the DG, as other hippocampal sub-regions appeared unaffected.

Aggrecan is an ECM component of the PNN, expressed in the hippocampus primarily around Parv+ interneurons (McRae et al., 2010). In order to confirm the reduced PNN immunoreactivity, aggrecan expression in the DG was evaluated. Reduced aggrecan immunoreactivity was also observed (Figure 4C), confirming the PNN depletion.

Interestingly, the percentage of Parv+ neurons ensheathed by lectin+ PNNs in the DG is significantly and progressively reduced from 6- to 12-months of age in TgproNGF#3 mice (Figure 4A), closely paralleling the reduction of total Parv+ interneurons (100% refers to the total amount of Parv+ cells in the DG; the histogram in red indicates the fraction of the total Parv+ neurons ensheathed by the PNN).

### Transcriptomic analysis in the hippocampus of TgproNGF#3 mice

**Figure 5:**
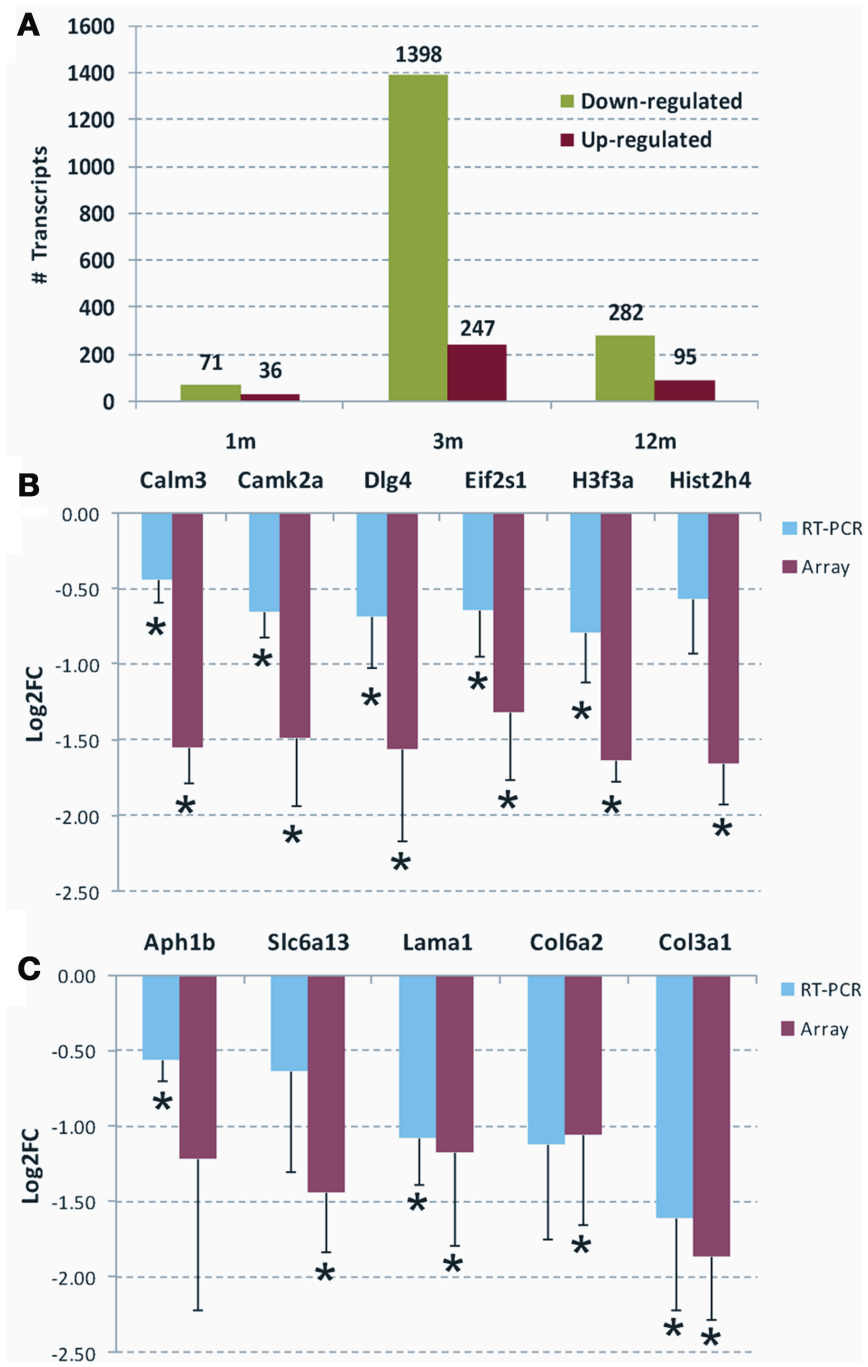
**(A)** Overall statistics of differentially expressed mRNAs in the hippocampus of 1-, 3-, and 12-months old TgproNGF#3 mice. Only transcripts with Limma *p* < 0.05 and |Log2 fold change| > 1.0 were selected. The graph shows the overall down-regulation of transcripts. **(B,C)** Validation of selected genes by qRT-PCR, respectively, at 3- and 12-months of age. The Log2 fold change (Log2 FC) ratio in microarray (red) and qRT-PCR (blue) are shown. The error bar is the *S.E.M*. and (*) indicates statistical significance by *T*-test (*P < 0.05).

In order to evaluate whether Parvalbumin interneuron/PNN depletion in the DG of TgproNGF#3 mice is sustained by a modulation of the expression of specific transcripts and gene families, we analyzed the hippocampal transcriptome of TgproNGF#3 mice at different ages (1-, 3-, and 12-months) by microarray analysis. Transcriptional changes are usually known to anticipate the onset of phenotypic alterations, therefore the expression profiling study was performed also at early age (1-month), when neurodegenerative traits and behavioral deficit are not established yet (Tiveron et al., 2013). In 1-month-old TgproNGF#3 mice, only a few mRNAs were differentially regulated (107 transcripts), with 34% up-regulated and 66% down-regulated (Figure 5A). Three-months-old TgproNGF#3 mice show a striking global and massive down-regulation of transcription (1645 transcripts modulated, only 15% up-regulated and 85% down-regulated) (Figure 5A). Similarly, in 12-month-old TgproNGF#3 mice (377 transcripts) 25.2% of differentially expressed mRNAs were found to be up-regulated and 74.8% mRNAs down-regulated (Figure 5A). Complete list of differentially expressed mRNAs at 1, 3 and 12 months is included in Supplementary Information Files as Table S1.

**Figure 6:**
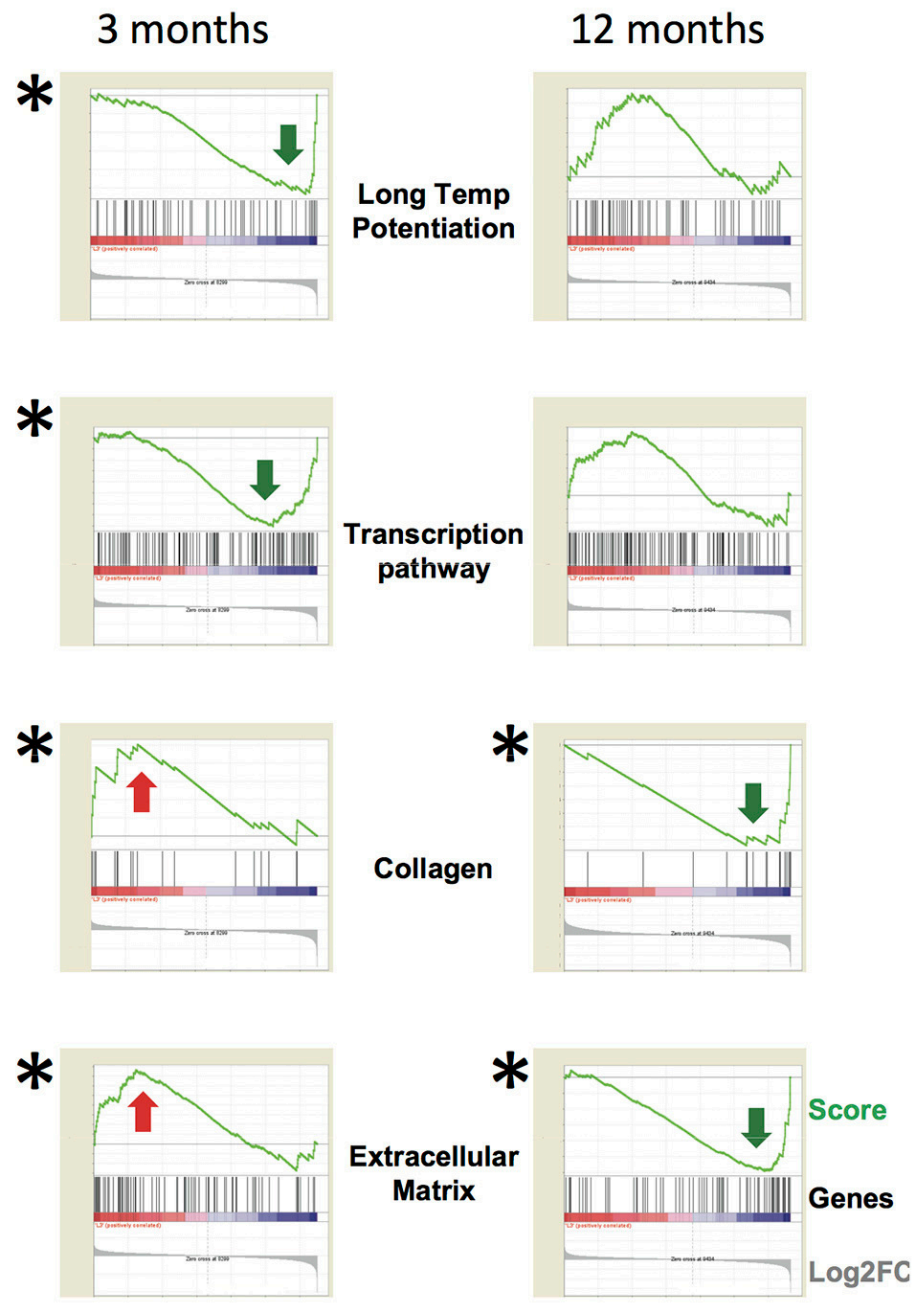
**Functional analysis of specific gene categories**. The whole dataset was analyzed by the tool GSEA (Gene Set Enrichment Analysis). FDR *q* < 0.05 (statistically significant) is indicated by (*) (obtained by gene set permutation). The GSEA analytical tool allows to identify small changes affecting a large set of functionally related genes compared to a *per gene* statistics. GSEA analytical tool allows to identify with high sensitivity global up- or down-regulation trends affecting large sets of functionally related genes. GSEA plots represent, for each functional category, a kind of summation integral of expression change for all single genes in the category. The methodology is sensitive even for small changes involving large gene sets. The GSEA plots highlight a general down-regulation of the Long Term Potentiation and Transcription systems at 3-months, while the Collagen and Extracellular Matrix categories are up-regulated at 3-months and down-regulated at 12-months. In each panel, the green line is the GSEA score plot, while the gray plot on the bottom of each panel is the global distribution of Log2 fold change for all genes, with up-regulated genes on the left and down-regulated ones on the right. The black thin bars on the bottom of each sub-panel indicate the genes belonging to the functional category, with each bar position corresponding to the specific score and Log2 fold change levels in the two plots. The following link provides information about how to interpret GSEA data and plot: http://software.broadinstitute.org/gsea/doc/GSEAUserGuideFrame.html?Interpreting_GSEA.

Clustering of the differentially expressed genes into functional categories, by gene-ontology tools including DAVID and GSEA analysis (Table 1 and Figure 6), shows that:

**(i)** TgproNGF#3 hippocampus has a clear proNGF-induced transcriptional signature (D'Onofrio et al., 2011; Arisi et al., 2014), markedly different from the response typically evoked by NGF, which is characterized by a strong induction of transcripts; no NGF-response genes were induced in the hippocampus of TgproNGF#3 mice (see list in Table S2).
**(ii)** At 1-month of age, mRNAs encoding proteins involved in synaptic transmission, such as synaptotagmin X (Syt10), and an ECM component proteoglycan (Prg4) were down-regulated, early representatives of two major gene families globally regulated at later ages (Table 1).
**(iii)** In 3-month-old TgproNGF#3 mice hippocampus, the most significantly down-regulated categories of mRNAs include
  – transcription, chromatin packaging and remodeling
  – synaptic transmission, LTP, glutamatergic transmission (Table 1, validation of selected individual mRNAs in Figure 5B)
  Conversely,
  – the up-regulation of ECM genes stands out in contrast to this general scenario of down-regulated transcription.

**(iv)** Interesting findings derive from GSEA analysis (Figure 6). At 1-month functional analysis reveals down-regulation of the Long Term Depression mRNA system, while the ECM gene sets are up-regulated. At 3-months the GSEA plots highlight a general down-regulation of the Long Term Potentiation and Transcription systems, while the Collagen and ECM categories are up-regulated at 3 and down-regulated at 12-months. GSEA analytical tool allows identifying with high sensitivity global up- or down-regulation trends affecting large sets of functionally related genes. GSEA plots represent, for each functional category, a kind of summation integral of expression changes for all single genes in the category. The methodology is sensitive even for small changes involving large gene sets. Such findings were confirmed also using a second well-established tool, DAVID, recently updated, for gene Ontology and pathway analysis. These methodologies are also robust to a reasonable rate of false positive differential genes. Both tools reach very similar biological conclusions, though being based on different algorithms, which further support the findings of the study.

**Table 1:** **Comparative functional analysis of differentially expressed mRNAs in the hippocampus of 3- vs. 12-month-old TgproNGF#3 mice**.

**Category**	**Term**	**Up 3 m**	**Down 3 m**	**Up 12 m**	**Down 12 m**
GOTERM_CC_4	Proteinaceous extracellular matrix	5.68E-04	.	.	7.45E-10
PANTHER_MF_ALL	Extracellular matrix	2.11E-03	.	.	4.03E-10
GOTERM_CC_4	Extracellular matrix part	6.02E-03	.	.	2.93E-05
SP_PIR_KEYWORDS	extracellular matrix	7.43E-03	.	.	1.96E-09
PANTHER_PATHWAY	Integrin signaling pathway	1.58E-03	.	.	2.92E-03
PANTHER_FAMILY	COLLAGEN ALPHA CHAIN	2.37E-02	.	.	1.55E-05
INTERPRO	Collagen triple helix repeat	2.52E-02	.	.	2.07E-05
SP_PIR_KEYWORDS	Collagen	2.99E-02	.	.	2.16E-05
SP_PIR_KEYWORDS	Cytokine	.	.	1.48E-02	.
GOTERM_BP_5	Induction of positive chemotaxis	.	.	2.54E-02	.
PANTHER_MF_ALL	Major histocompatibility complex antigen	.	.	.	9.09E-05
GOTERM_CC_5	MHC protein complex	.	.	.	2.93E-04
GOTERM_BP_4	Muscle tissue morphogenesis	3.71E-04	.	.	.
GOTERM_BP_5	Negative regulation of apoptosis	1.34E-02	.	.	.
SP_PIR_KEYWORDS	Phosphoprotein	.	1.80E-16	.	.
SP_PIR_KEYWORDS	Acetylation	.	1.08E-06	.	.
SP_PIR_KEYWORDS	Methylation	.	1.35E-06	.	.
GOTERM_CC_4	Microbody	.	1.77E-03	.	.
GOTERM_CC_4	Peroxisomal part	.	2.76E-03	.	.
GOTERM_BP_5	Regulation of gene expression	.	1.16E-04	.	.
GOTERM_BP_5	Regulation of transcription	.	1.14E-04	.	.
PANTHER_BP_ALL	Chromatin packaging and remodeling	.	1.62E-03	.	.
GOTERM_CC_4	Chromatin	.	2.43E-03	.	.
KEGG_PATHWAY	Long-term potentiation	.	3.41E-05	.	.
GOTERM_CC_4	Ionotropic glutamate receptor complex	.	9.02E-03	.	.
GOTERM_BP_5	Regulation of synaptic transmission	.	5.31E-05	.	.
GOTERM_CC_4	Neuron projection	.	1.25E-05	.	.
GOTERM_CC_4	Dendrite	.	8.54E-03	.	.

*The analysis of over-represented mRNA categories was performed using the DAVID tool. Only differential genes corresponding to each time points were used. P-values are shown for up and down-regulated genes in separate columns. Categories referring to genes related to collagen (yellow) and extracellular matrix (light blue) have opposite trends at 3-months compared to 12-months of age. Categories referring to similar genes have the same color*.

Such global analysis provides therefore interesting and significant results.

At 12-months, down-regulation of aggrecan transcript, a PNN component, is detected by microarray and highlighted by GSEA analysis (Figure 6), in line with the reduced aggrecan protein expression observed by immunofluorescence (Figure 4C).

**(v)** Significantly, gene categories related to collagen and ECM have opposite trends at 3-months (up-regulated) and 12-months of age (down-regulated, Table 1). At 12-months we observed a significant down-regulation of collagens and PNN (a condensation of ECM proteins ensheathing Parvalbumin interneurons) components.
**(vi)** Notably, no reduction in global hippocampal GAD transcript was observed in 3- and 12-month-old TgproNGF#3 mice.

### Validation by real time qRT-PCR of a selected panel of transcripts

Chloride transporters Nkcc1 and Kcc2, known determinants of E/I homeostasis, essential in establishing GABAergic system maturation, were not found to be modulated in microarray analysis. In order to confirm this data, the mRNA expression of chloride transporters Nkcc1 and Kcc2 in TgproNGF#3 mice hippocampus was evaluated by real-time RT-PCR at 1.5, 3, and 12-months of age. No significant change in the expression pattern was observed (Figure S6). Maturation and function of parvalbuminergic Fast Spiking interneurons critically depend on BDNF (Berghuis et al., 2004; Cancedda et al., 2007). However, microarray analysis showed that global BDNF expression was unchanged in the hippocampus of TgproNGF#3 mice. In order to confirm the lack of variation, the global level of BDNF-encoding mRNA was evaluated by real time qRT-PCR. No major changes were observed (not shown). Several differentially regulated transcripts highlighted by gene-ontology analysis were individually validated by real time qRT-PCR, confirming the down-regulated pattern (Figures 5B,C): at 3-months, calmodulin3 (Calm3), calcium/calmodulin-dependent protein kinaseII alpha (CAMK2a), PSD95 synaptic protein [discs large homolog 4 (Drosophila) or Dlg4] and the translation initiation factor eIF2, at 12-months, collagen, type III alpha 1 (Col3a1), collagen, type VI alpha 2 (Col6a2), laminin, alpha 1 (Lama1) (Chung et al., 2005; Cheng et al., 2009; Dityatev et al., 2010; Mercier and Arikawa-Hirasawa, 2012). Finally, at 12-months some components of the ion channels/transporters gene family are modulated, such as the GABA transporter solute carrier family 6 (Slc6a13, also known as GAT2) (Zhou and Danbolt, 2013). Interestingly, the trend observed in array analysis was confirmed by real time qRT-PCR, often in a significant pattern. A few of the modulated transcripts could not be significantly validated by real time qRT-PCR. However, bioinformatic tools for gene Ontology and pathway analysis (GSEA and DAVID) highlighted the significant involvement of ECM among the modulated categories.

### Expression of BDNF-encoding mRNAs: selective modulation of BDNF transcript variants

Having established that the parvalbuminergic interneuron population is selectively reduced in the DG of TgproNGF#3 mice, we investigated the possible role of the BDNF neurotrophin. The role of BDNF in interneuron maturation and function is well-established (Huang et al., 1999; Cancedda et al., 2007; Sakata et al., 2009), however in TgproNGF#3 mice the global level of BDNF-encoding mRNAs in the hippocampus was unaffected, as revealed by microarray analysis.

The rodent BDNF gene produces several different splicing variants (Figure S7), each composed of one alternatively spliced 5′UTR exon linked to a common downstream exon containing the coding region with two possible (either short or long) 3′UTRs (Aid et al., 2007). Therefore, we further analyzed the expression of individual BDNF transcript variants by qRT-PCR.

**Figure 7:**
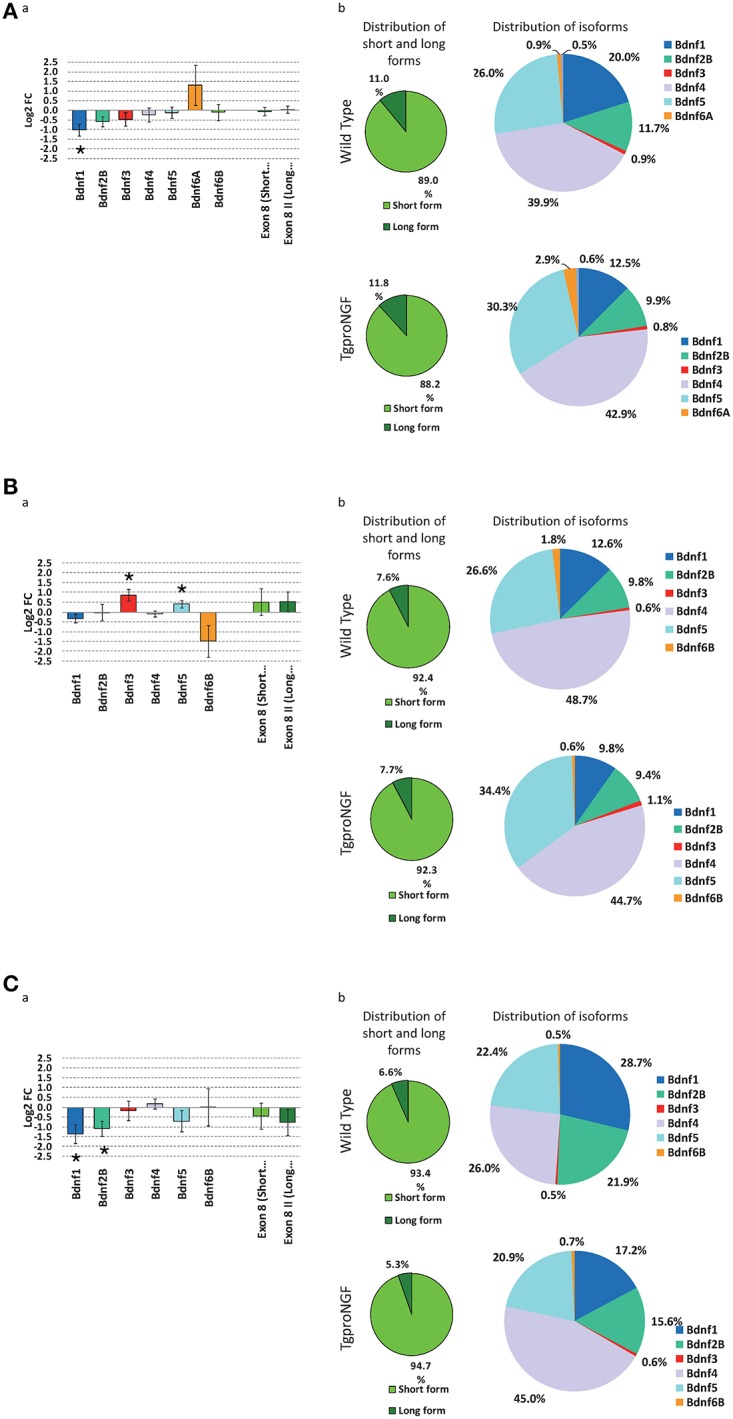
**(A)** Expression level of Bdnf gene isoforms, by qRT-PCR, at 1-month of age. **(a)** Differential expression of main Bdnf isoforms, measured ad Log2 fold change ratio TgproNGF#3 vs. Wild Type; (*) indicates a statistical significant variation by 1-tail *T*-test for Bdnf1, while the two rightmost bars indicates the global relative expression of short and long isoforms. **(b)** Relative distribution of short and long Bdnf forms (left) and of the different isoforms (right) in the TgproNGF#3 and Wild Type mice: the relative proportion of short and long isoforms is comparable in the two mouse strains. **(B)** Expression level of Bdnf gene isoforms, by qRT-PCR, at 3-months of age. **(a)** Differential expression of main Bdnf isoforms, measured ad Log2 fold change ratio TgproNGF#3 vs. Wild Type; (*) indicates a statistical significant variation by 1-tail *T*-test, for Bdnf3 and Bdnf5, while the two rightmost bars indicates the global relative expression of short and long isoforms. **(b)** Relative distribution of short and long Bdnf forms (left) and of the different isoforms (right) in the TgproNGF and Wild Type mice: the relative proportion of short and long isoforms is comparable in the two mouse strains. **(C)** Expression level of Bdnf gene isoforms, by qRT-PCR, at 12-months of age. **(a)** Differential expression of main Bdnf isoforms, measured ad Log2 fold change ratio TgproNGF#3 vs. Wild Type; (*) indicates a statistical significant variation by 1-tail *T*-test, for Bdnf1 and Bdnf2B, while the two rightmost bars indicates the global relative expression of short and long isoforms. **(b)** Relative distribution of short and long Bdnf forms (left) and of the different isoforms (right) in the TgproNGF#3 and Wild Type mice: the relative proportion of short and long isoforms is comparable in the two mouse strains.

In 1-month-old TgproNGF#3 mice, a significant down-regulation of BDNF transcript I was observed (Figure 7Aa)

Conversely, at 3-months of age a significant up-regulation of BDNF splice variants (III and V) was detected in TgproNGF#3 mice (Figure 7Ba).

At 12-months of age a significant down-regulation of BDNF splice variants 1 and 2b was detected in TgproNGF#3 mice (Figure 7Ca).

At 1-, 3-, and 12-months the relative proportion of short and long isoforms, regardless of their 5′ splicing pattern, was, on the other hand, unchanged in the two mouse strains (wt and transgenic, Figures 7A–C). The distribution of the 3′ short form, evaluated using primers encompassing the common coding exonVIII, confirmed that global BDNF levels were unaffected, as revealed by microarray (Figures 7A–C).

Therefore, the observed subtle specific changes in the expression of selected BDNF transcripts might underlie differential BDNF influence on interneuron maturation and homeostasis in TgproNGF#3 mice hippocampus.

### Early spontaneous epileptic-like events in TgproNGF#3 mice

**Figure 8:**
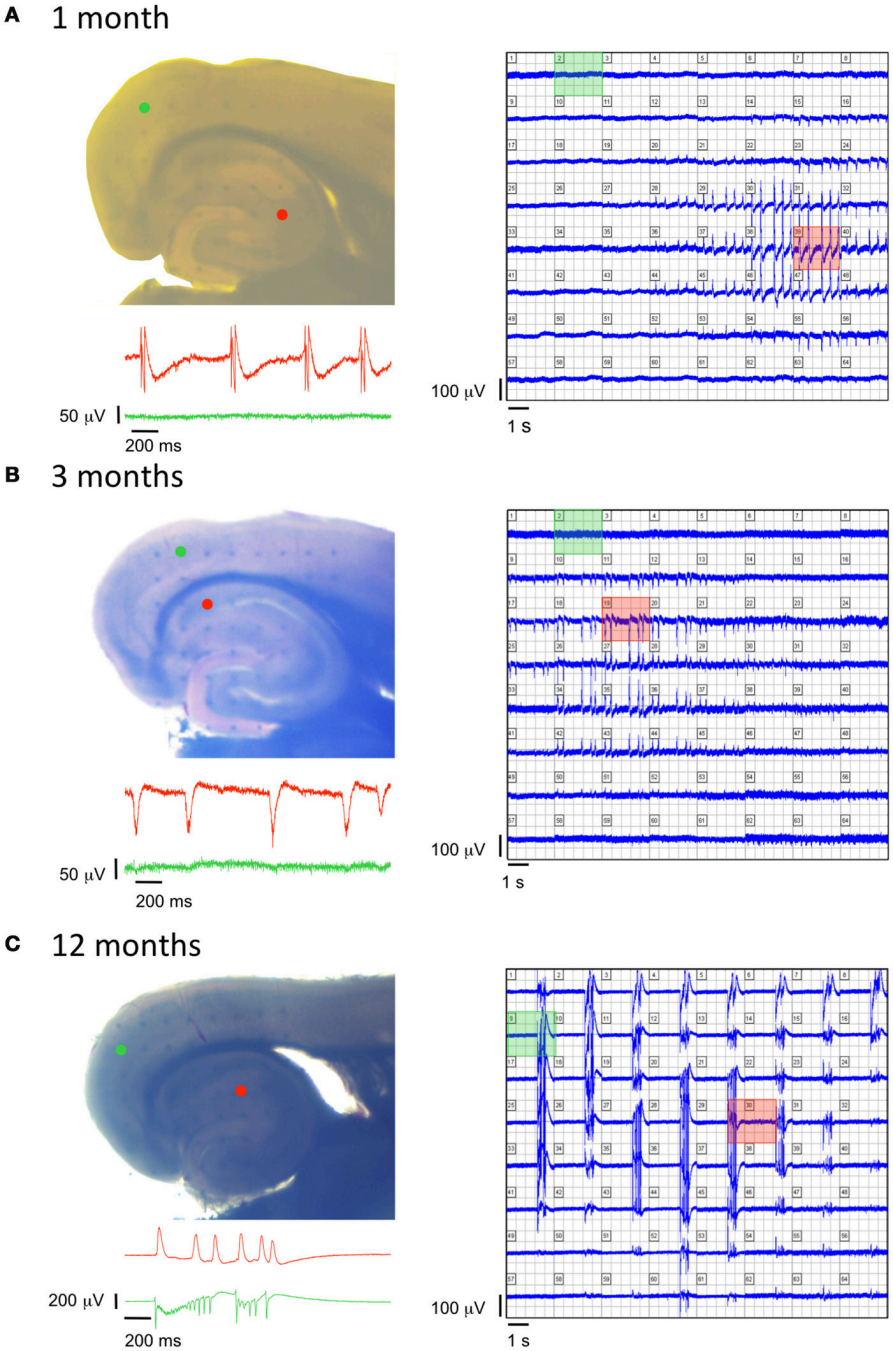
**Age-dependent modifications of epileptiform activity in TgproNGF#3 mice. (A–C** left panels) Photographs of EC-HP slices obtained from a 1-, 3-, and 12-months old TgproNGF#3 mouse, respectively, placed over an array of 64 planar multi-electrodes, detectable in transparency through the slice. Colored dots indicate position of the electrodes, whose voltage signals appear in the corresponding colored area. (**A–C** right panels) Simultaneous recordings from each one of the 64 electrodes. (**A–C** insets) Trace records from the HP (red) and EC (green) detected by the colored electrodes in the corresponding photograph, showing no spontaneous activity in the EC of 1- and 3-months old mice, as opposed to spontaneous cortical events at 12-month, while the hippocampal area displays spontaneous events at all 3 ages.

Aged TgproNGF#3 mice were previously shown to display spontaneous epileptiform interictal-like discharges in the entorhinal cortex-hippocampal (EC-HP) network, suggesting a role of proNGF/NGF balance in the E/I homeostasis (Tiveron et al., 2013). The age-dependency of this phenomenon was investigated by multielectrode analysis with field recordings, using a 64-channels MEA device, revealing the presence of spontaneous epileptic-like seizures in 1-, 3-, and 12-months-old TgproNGF#3 mice, in combined EC-HP slices. Repetitive spontaneous interictal-like events restricted to the hippocampal region were detected in every slice (*n* = 10) from 1-month-old TgproNGF#3 mice, while no spontaneous events were detected in the entorhinal area at this age (Figure 8A). Similarly, in slices (*n* = 5) of 3-months-old TgproNGF#3 mice, spontaneous interictal-like events were never observed in the EC, as opposed to a pronounced hippocampal spontaneous hyperactivity (Figure 8B). Conversely, slices from 12-month-old TgproNGF#3 mice displayed spontaneous interictal-like events not only in the hippocampus, but also in the entorhinal area (Figure 8C), in line with previous reports (Tiveron et al., 2013). Thus, TgproNGF#3 mice display spontaneous epileptic-like events in EC-HP slices starting from 1-month of age, well before the onset of behavioral and neurodegenerative changes.

## Discussion

The presence of spontaneous epileptic-like events in the HP/EC network, previously described in aged TgproNGF#3 mice (Tiveron et al., 2013), suggested the occurrence of an E/I imbalance, as a consequence of proNGF increase. In order to gain further insights into the mechanisms triggering and sustaining TgproNGF#3 mice phenotype, we investigated early events driven by proNGF/NGF imbalance in these mice.

Our results show that proNGF overexpression alters the interneuron-PNN-system, in a region- and interneuron subpopulation-selective manner. The DG appears to be particularly sensitive to the effects of proNGF.

### Parvalbuminergic interneurons are selectively depleted in the DG

Selective depletion of parvalbuminergic interneurons was detected in the DG, starting from 6-months of age, despite of no detectable enrichment in proNGF expression in this hippocampal sub-region. Interestingly, other hippocampal areas were preserved.

Selective depletion of Parv+ neurons was observed also in the basolateral amygdala, a region involved in the active avoidance response (Li and Richter-Levin, 2012), severely impaired in TgproNGF#3 mice (Tiveron et al., 2013). The amygdala, a brain region involved in memory processing, particularly in encoding emotional significance of environmental stimuli, strongly connected to other brain structures, receives, in analogy to the pyramidal cells and GABAergic interneurons of the hippocampus, cholinergic inputs from basal forebrain synapses that modulate both excitatory and inhibitory synaptic transmission (Feduccia et al., 2012). The cholinergic deficit previously described in TgproNGF#3 mice (Tiveron et al., 2013), and confirmed in the present study by transcriptomic data, may therefore affect both regions.

In both DG and amygdala alterations of parv interneurons are reported as consequence of stress, with opposite changes in the two regions affecting excitatory and inhibitory components in a homeostatic balance (Seidel et al., 2008).

Moreover, a number of studies have shown the role of amygdala Parv+ interneurons in fear conditioning and in active avoidance. Parvalbumin interneurons play a default-silencing role in the amygdala during fear memory encoding. When animals acquire a fear memory, the suppressive influence of Parv+ interneurons is relieved, allowing the fear system to respond promptly (Lucas et al., 2016). Parv+ interneuron inhibition shapes the size of neuronal memory ensembles (engram that allows patterns of activity present during learning to be reactivated in the future, Morrison et al., 2016). These findings suggest that, in case of Parv depletion, the basal default silencing activity of Parv+ cells might be disrupted and the aberrant lack of suppression of basal activity might, conversely, affect the encoding of fear memory.

In addition, during acquisition and extinction of an active avoidance task, neural activity in amygdala involves Parv+ interneurons, as revealed by induction of the immediate early gene product c-Fos (Jiao et al., 2015).

Therefore, the observed Parv+ depletion in basolateral amygdala is likely to contribute to the behavioral deficit in avoidance response observed in TgproNGF#3 mice.

The GABAergic Parv+ neurons are surrounded by the PNN (Pizzorusso et al., 2002). In post-natal development, the PNN restricts plasticity at the end of the critical period in the visual cortex and regulates firing of Parv+ neurons, influencing their maturation (Pizzorusso et al., 2002).In the DG of TgproNGF#3 mice, also the percentage of Parv+ neurons ensheathed by PNNs is significantly and progressively reduced. Enhanced neuronal activity is known to cause a decrease in the expression of components of the PNN (McRae et al., 2012), therefore such finding might be related to the aberrant spontaneous epileptiform activity in the EC-HP network. A deteriorated PNN surrounding Parv+ interneurons lays the ground for extrasynaptic movement of receptors and neurotransmitters into the extrasynaptic space (McRae and Porter, 2012), leaving inhibitory interneurons susceptible to increased synaptic reorganization (McRae et al., 2012).

Possible impairment of adult neurogenesis must be taken into account, since intracerebral injection of proNGF inhibits SGZ neurogenesis (Guo et al., 2013). Interestingly, among modulated RNAs there is laminin, alpha 1 (Lama1), an ECM protein component of PNN and structural components of basal laminae found in the fractones (Mercier and Arikawa-Hirasawa, 2012) of the neurogenic niche and contributing to synapse formation (Dityatev et al., 2010).

Moreover, it will be interesting to evaluate, in future analysis, a possible impairment in electrical oscillation patterns in these mice, since Parv+ interneurons are the fast-spiking (FS) population driving oscillation rhythms, including gamma oscillations (Buzsáki and Wang, 2012).

It is known that BDNF crucially controls the functional differentiation and maturation of FS-interneurons (Berghuis et al., 2004); surprisingly the overall expression of BDNF transcript was unchanged in TgproNGF#3 mice hippocampus. However, more subtle changes in selective splicing isoforms of BDNF have been detected in TgproNGF#3 mice (see below).

The selective involvement of the dentate gyrus (DG) deserves some comments. In neurodegenerative models the DG is often selectively compromised (Palop et al., 2005). The DG represents a crossroad, receiving the perforant path as main excitatory input, which funnels distinctly unidirectional progression of excitatory activity arriving from other brain regions to the trisynaptic hippocampal circuit. These excitatory synaptic inputs are complemented by cholinergic, GABAergic, noradrenergic, dopaminergic, and serotonergic projections (Perederiy and Westbrook, 2013). The adult brain is in a continuous state of remodeling. This is particularly true in the DG, where competing forces, such as neurodegeneration and neurogenesis, dynamically modify neuronal connectivity and can occur simultaneously (Perederiy and Westbrook, 2013). Altered or aberrant activity in such a critical node might alter the excitation/inhibition balance in the DG (as it is known to occur after lesion of the perforant path, Clusmann et al., 1994).

### Calbindin depletion in DG granule cells

In neurodegeneration models Ca^++^ buffer proteins are often altered in the DG: hAPP mice (a mouse model of AD) develop AD-like abnormalities, including depletions of calcium-related proteins in the DG, spontaneous non-convulsive seizure activity in cortical and hippocampal networks, associated with synaptic plasticity deficits in the DG (Palop et al., 2007). Moreover, epileptiform activity can also lead to depletion of calcium-dependent proteins over time (Palop et al., 2011). Calbindin+ neurons were therefore evaluated in the same region.

In aged TgproNGF#3 mice, a marked depletion of calbindin (CB)-protein in granule cells and in their axons projecting to CA3 (Mossy fibers) was observed. Recent evidence that removal of CB from amyloid precursor protein/presenilin transgenic mice aggravates AD pathology, suggests a critical role of CB (Kook et al., 2014). Moreover, in late-stage AD, a higher ratio of CB-negative granule cells is detected in the DG (Stefanits et al., 2014). In TgproNGF#3 mice we previously described a learning and memory deficit starting from 3-months of age (Tiveron et al., 2013). The late calbindin deficit observed in the DG may contribute to aberrant neuronal activity. Evaluation of DG-LTP will be necessary in order to investigate a selective plasticity deficit. No LTP deficit was previously detected in CA1 (Tiveron et al., 2013).

### Hippocampal transcriptional profiling reveals a specific proNGF-induced signature, lacking NGF-response genes induction, with broad early down-regulation of transcripts

The most remarkable finding of transcriptome analysis is the global down-regulation of mRNAs expression in the hippocampus of TgproNGF#3 mice in early neurodegeneration (at 3-months), a trend that already begins at 1-month. Consistently, a broad reduction in the expression of genes involved in regulation of transcription and chromatin remodeling is observed. Notably, the expression of mRNAs known to be heavily regulated by NGF (Egr1, Egr2, Egr4, Fos, Jun, Arc, Myc, Vgf) (whose induction represents, on the contrary, a typical “NGF signature,” see Dijkmans et al., 2009), is not changed in TgproNGF#3 mice hippocampus, despite the presence of mature NGF, consequent to cleavage by extracellular proteases, as previously measured (Tiveron et al., 2013). This confirms that proNGF signaling predominates over that of NGF in these mice, confirming what had been shown in cellular systems exposed to either NGF or proNGF added singularly or in various combinations (D'Onofrio et al., 2011; Arisi et al., 2014).

Notably, hippocampal transcriptional profiling reveals, a clear dominant “proNGF signature,” with broad down-regulation of transcription, whereas lacks completely the classical “NGF signature,” characterized typically by IEG induction, followed by an up-regulation of their target genes (D'Onofrio et al., 2011).

### Gene ontology analysis highlights marked down-regulation of synaptic transmission-related genes

Down-regulated transcript categories include those related to synaptic transmission and synaptic plasticity, such as Calm3 (principal mediator of Ca^++^ signal, essential for CAMK activation), CAMKIIa, Dlc4 (better known as PSD95, main constituent of the post-synaptic compartment, essential for spine stability) and EIF2, involved in translational control of synaptic plasticity, acting on local protein synthesis (Toutenhoofd and Strehler, 2002; Bingol et al., 2010; Borck et al., 2012; Cane et al., 2014). Interestingly functional analysis of differential categories reveals down-regulation of Long Term Depression system at 1-month and of Long Term Potentiation at 3-months. It would be interesting to evaluate in future electrophysiological studies both LTD and LTP in this model.

Specific effects of proNGF on such crucial protein targets, essential for synaptic plasticity and for the establishment of long-term memory, may well sustain the behavioral deficits previously described in this model (Tiveron et al., 2013).

In TgproNGF#3 mice the observed spontaneous epileptic-like discharges could be due, in principle, to changes in post-natal maturation of Cl^−^ homeostasis, known to determine GABAergic system post-natal maturation. However, the expression of the cation-chloride cotransporters Nkcc1 and Kcc2 mRNA was not significantly altered in TgproNGF#3 mice HP. The most interesting insights of expression profiling derive from the analysis of the globally modulated categories, more than of single transcripts: LTP—related or synaptic transmission—related transcripts and ECM, as described below.

### ECM components show initial up-regulation followed by late down-regulation

In aged transgenic mice fewer genes were differentially modulated. The most striking transcriptional change involves ECM transcripts: interestingly, the trend is opposite in early and late neurodegeneration. Initial up-regulation of ECM transcripts at 3-months, possibly contributing to impaired plasticity, stands out in the context of a general mRNA down-regulation scenario, and is followed by their severe down-regulation at 12-months. Human genetic studies and analysis of transgenic mice deficient in ECM molecules link ECM molecules to epileptogenesis (Suzuki et al., 2002; Dityatev, 2010; Geissler et al., 2013). Our results place proNGF as a regulator of this link.

Interestingly, a consistent down-regulation of a number of collagen mRNAs, alongside the regulation of a number of ECM related mRNAs was observed in TgproNGF#3 mice, a finding which could be mechanistically related to structural alterations in the GABAergic inhibitory network and to a disruption of the excitatory/inhibitory balance. Indeed, the ECM is at the crossroad of circuit development, reshaping synaptic plasticity and excitatory/inhibitory balance in the nervous system. It is tempting to suggest that the ECM dysregulation uncovered by this transcriptional profile in TgproNGF#3 mice might be related to the neurological aspects of matrix diseases. Further studies will be required to investigate this aspect further.

### Alterations in the expression of selected BDNF splice variants

Taking into account that BDNF crucially controls the functional differentiation and maturation of FS-interneurons (Berghuis et al., 2004; Cancedda et al., 2007) we evaluated changes in the expression of BDNF splice-variants pattern.

BDNF splice variants are characterized by differential distribution in brain regions and in neuronal subcellular compartments (somatic vs. dendritic); their pattern is modulated in response to various stimuli and activity-dependent BDNF mRNA localization in dendrites is observed. Splice variant patterns constitute therefore a “spatial and temporal code” directing BDNF expression locally. Such regulation explains the contradictory effects of BDNF, which may oppose or promote epileptogenesis (Tongiorgi et al., 2006; Chiaruttini et al., 2008; Sakata et al., 2009). In the hippocampus, in basal conditions, the main BDNF dendritic variant is BDNF6 (although other variants are expressed in smaller amounts: 7 in CA1; 1, 6, and 9a in CA3; and 5, 6, 7, and 8 in DG). Stimuli such epileptogenesis prompt dendritic accumulation of variants 4 and 6 (and also, in small amounts, in selected subregions, of BDNF2, 3, and 9a) (Chiaruttini et al., 2008; Baj et al., 2013). Interestingly, in prefrontal cortex BDNF4 is known to play an essential role in GABA interneuron homeostasis (Sakata et al., 2009). Notably, proNGF selectively alters the complex pattern of BDNF transcripts, up-regulating BDNF3 and BDNF5 splice variants in 3-month-old TgproNGF#3 mice hippocampus. In the brain, BDNF3 is the only splice variant also expressed by microglial cells (Kruse et al., 2007) and is known to participate in macrophages activation process in an autocrine manner (Asami et al., 2006). The up-regulation of isoform BDNF3 at 3-months, suggests therefore an interesting involvement of microglial signaling. Future characterization of microglial phenotype is therefore necessary to address their hypothesized role. It is known that pilocarpine-treatment inducing status epilepticus changes the pattern of hippocampal BDNF mRNA variants in rat, selectively increasing transcript encoding exon3 in the DG (Baj et al., 2013). Notably, BDNF3 up-regulation is also induced by endothelin1 (ET1) (Böhm and Pernow, 2007), whose intraventricular injection evokes epileptic seizures, apparently mediated by its vasoconstrictor effect (Koyama et al., 2005). The hypothesis that up-regulation of BDNF3 in microglial cells, as a consequence of proNGF/NGF imbalance, contributes to E/I imbalance is intriguing and need to be addressed in future studies.

Consequences of up-regulation of BDNF5 (at 3-months of age) and down-regulation of BDNF1 (at 1- and 12-months) and BDNF2b (at 12-month), also observed in TgproNGF#3 mice hippocampus, require further investigation.

### Spontaneous epileptiform discharges in the HP network are a very early event

We observe the presence of spontaneous epileptic-like events in the HP network, previously described in aged TgproNGF#3 mice, in the hippocampus as early as at 1-month of age, when (as reported in Tiveron et al., 2013), the cholinergic deficit is not established yet, but proNGF is already accumulating. These spontaneous discharges spread also to the EC network at later ages (12-months). Therefore, their onset precedes the learning and memory deficit observed at 3-months of age, suggesting that E/I imbalance is a primary and direct consequence of proNGF/NGF unbalanced signaling. However, TgproNGF#3 mice do not exhibit frank spontaneous seizures; possible increased susceptibility to seizures will be evaluated in the future.

### Cellular targets of proNGF action

The known cellular targets of proNGF action are cells expressing p75^NTR^. Hippocampal expression of p75^NTR^ in principal neurons is weak or absent (Dougherty and Milner, 1999).

**Figure 9:**
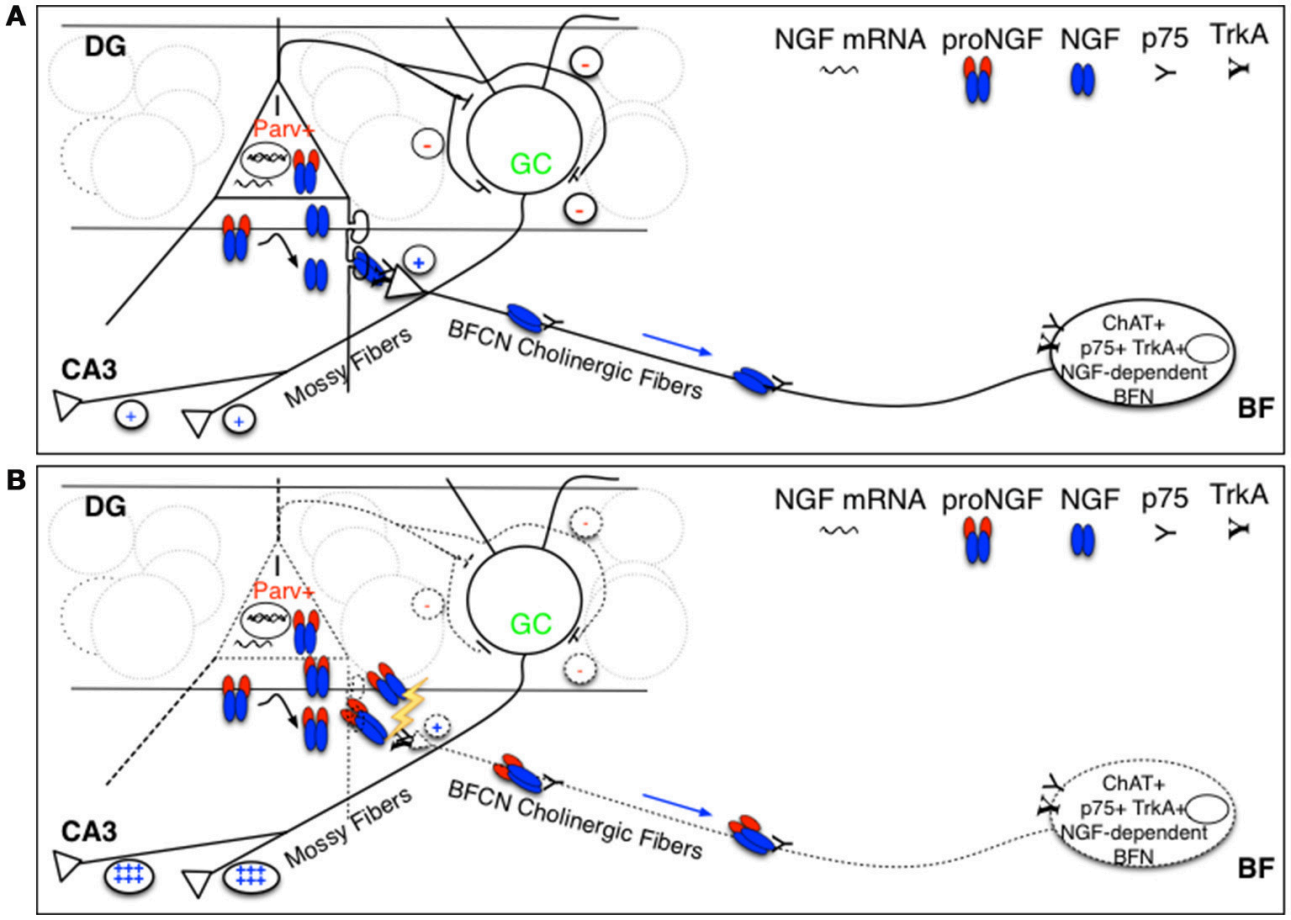
**Schematic representation of hypothesized targets of proNGF/NGF imbalance. (A)** We hypothesize that proNGF/NGF imbalance primarily impacts Parv+ interneurons that make inhibitory synaptic contact with DG granule cells that project their axons (the so-called Mossy fibers) to CA3 neurons. These interneurons normally produce NGF and feed cholinergic fibers deriving from NGF-dependent BFN expressing p75NTR, making synaptic contact with Parv interneuron dendrites in the DG. In such a way, cholinergic input tunes Parv+ interneuron inhibitory output onto granule cells, affecting their excitatory activity to CA3 neurons (mediated by Mossy fibers). **(B)** Overexpression of furin-resistant proNGF, producing an NGF/proNGF imbalance, severely impairs cholinergic fibers, disrupting this feed-back control mechanism influencing hippocampal memory encoding and retrieval and contributing to E/I imbalance. I, interneuron; Parv+, parvalbuminergic interneuron; GC, granule cell; DG, dentate gyrus; BFN, Basal Forebrain Neuron.

Parv+ interneurons in the DG do not express p75^NTR^, whereas they are physiologically known to express NGF (Holm et al., 2009; Biane et al., 2014) and to feed the NGF-dependent BF cholinergic fibers, expressing both TrkA and p75^NTR^, that make synaptic contacts with them in the DG (Figure 9), predominantly with processes (Dougherty and Milner, 1999; Ludkiewicz et al., 2002). Parv+ interneurons do not express trkA, but are known to express trkB, being responsive to BDNF (Holm et al., 2009). However, the overall expression of BDNF is not affected in TgproNGF#3 mice hippocampus. Interestingly, some changes in BDNF splicing variants were observed: a different pattern in BDNF splicing variants may account, at least in part, for alterations in Parv+ interneurons homeostasis. A cholinergic deficit starting from 3-months was detected in TgproNGF#3 mice (Tiveron et al., 2013) as confirmed also by hippocampal transcriptomic data (not shown). Pathological conditions characterized by increased levels of proNGF in the brain, might lead to a reduced cholinergic drive to Parv+ interneuron, with the ensuing E/I imbalance (Figure 9B). How astrocytes, which are also a potential target of proNGF actions, contribute to this mechanism is being currently investigated.

Taking into account the time-scale of the observed cholinergic deficit (measured as reduced counting of ChAT+ neurons, starting from 3-months of age, Tiveron et al., 2013), it precedes the selective depletion of Parv+ interneurons. However, it seems difficult to explain the early onset of epileptiform discharges in the hippocampus (at 1-month of age), as a consequence of an interneuron dysfunction occurring much later, according to the timeline of progression of TgproNGF#3 phenotype. Moreover, we previously showed (Tiveron et al., 2013) that cholinergic impairment is not present at 1-month of age, therefore it cannot represent the “primum movens” responsible of the supposed hyperexcitability suggested by MEA results, although we cannot exclude that a reduced functional cholinergic drive begins even earlier.

To better understand the hypothesized cycle linking proNGF, neurodegeneration and E/I homeostasis, further electrophysiological evaluation at early stages of proNGF/NGF imbalance is needed. A functional study directly addressing the effective E/I ratio is therefore expected to provide more clues about the real upstream driver.

Moreover, the selective involvement of the DG in alterations of Parv+ neurons/PNN system, jointly with transcriptomic data, showing down-regulation of synaptic transmission-related transcripts, suggest future analysis of DG–LTP, frequently altered in neurodegeneration models (Houeland et al., 2010).

## Conclusions

Neuronal network hyperexcitability and cognitive dysfunction have been detected in mouse models of AD and associated to depletion of Ca^++^ dependent proteins and to inhibitory interneuron deficits or hippocampal remodeling (Palop et al., 2011; Verret et al., 2012). Reported experimental evidence favors a view of E/I imbalance as a consequence of amyloidogenic process activation (Palop and Mucke, 2009; Palop et al., 2007; Palop and Mucke, 2010; Harris et al., 2010), but also as upstream driver of neurodegeneration and cognitive impact (Sanchez et al., 2012), since antiepileptic treatment was shown to slow down the cognitive decline in transgenic models of neurodegeneration and, recently, in MCI subjects (Sanchez et al., 2012; Bakker et al., 2015).

Our results provide further mechanistic insights into the negative cycle linking proNGF, neurodegeneration and E/I homeostasis (Tiveron et al., 2013).

In summary, we observed a regional- and cellular-selective Parvalbumin interneuron and PNN depletion in the DG, but not in other hippocampal regions. These results demonstrate that in the hippocampus the DG is selectively vulnerable to altered proNGF/NGF signaling. Parvalbumin interneuron depletion is also observed in the amygdala, a region strongly connected to the hippocampus and likewise receiving cholinergic afferences.

However, the onset of spontaneous discharges in the hippocampal network precede those changes, suggesting E/I imbalance represents a primary event, immediately following proNGF accumulation. The observed alterations in the expression of selected BDNF splice variants might be directly involved; the impact of those changes on E/I balance and how they relate to proNGF increase are intriguing questions that need to be addressed further.

## Author contributions

LF and RB are joint first authors. LF, RB, and AC: designed experiments. LF, RB, FL, IA, NB, MD, and AC: performed experiments. LF, RB, IA, FL, NB, SC, MD, and AC: analyzed data. LF and AC wrote the manuscript. All authors read, reviewed, and commented the manuscript. LF and IA prepared the figures.

## Funding

This work was funded by the following grants: FIRB RBAP10L8TY from the Italian Ministry of Higher Education and Scientific Research; Fondazione Roma; PAINCAGE FP7 Collaborative Project number 603191; Fondazione Italiana Sclerosi Multipla FISM 2013/R/6; Italian Research Council (Framework Agreement EBRI-CNR 2015–2017) and European grant Horizon H2020-ICT-2016, “MADIA,” number 1732678.

### Conflict of interest statement

The authors declare that the research was conducted in the absence of any commercial or financial relationships that could be construed as a potential conflict of interest.

## Abbreviations

NGF: Nerve Growth Factor
AD: Alzheimer's Disease
wt: wild-type
HP: hippocampus
BF: basal forebrain
BFCN: basal forebrain cholinergic neurons
EC: entorhinal cortex
E/I: excitatory/inhibitory
MEA: multi electrode array
ECM: extracellular matrix proteins
PNN: perineuronal net
Parv: Parvalbumin
CB: calbindin
MCI: mild cognitive impairment
DG: dentate gyrus
FS: fast-spiking
BDNF: brain-derived neurotrophic factor
LTP: Long Term Potentiation
LTD: Long Term Depression.
