# Supplementary material for: ProNGF Drives Localized and Cell Selective Parvalbumin Interneuron and Perineuronal Net Depletion in the Dentate Gyrus of Transgenic Mice
Source: Front Mol Neurosci. 2017 Feb 9;10:20. doi: 10.3389/fnmol.2017.00020 (PMC5299926; doi:10.3389/fnmol.2017.00020)
Supplement: Supplementary file 2 [file Presentation1.pptx]

## Slide 1
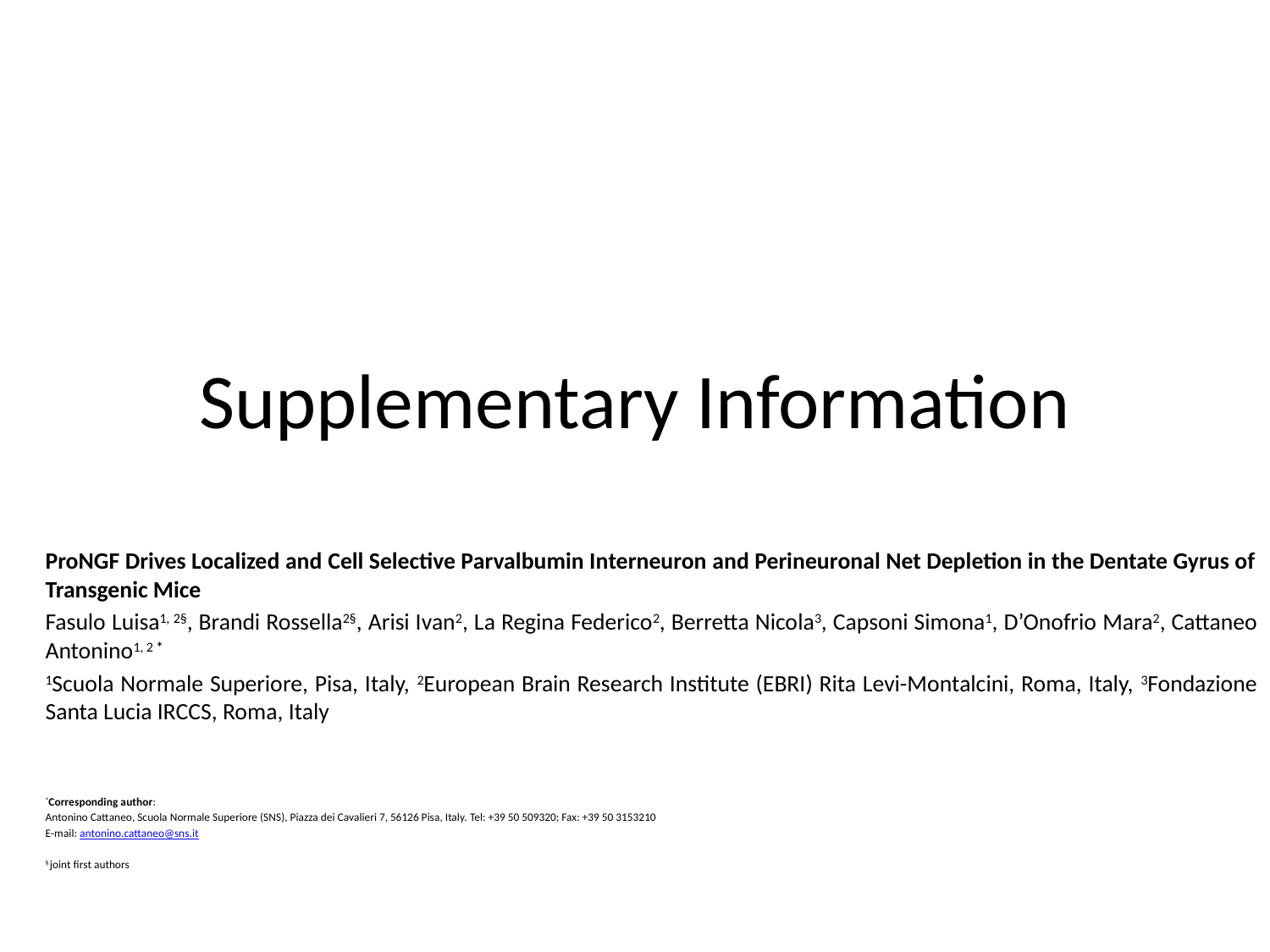

# Supplementary Information
ProNGF Drives Localized and Cell Selective Parvalbumin Interneuron and Perineuronal Net Depletion in the Dentate Gyrus of Transgenic Mice
Fasulo Luisa1, 2§, Brandi Rossella2§, Arisi Ivan2, La Regina Federico2, Berretta Nicola3, Capsoni Simona1, D’Onofrio Mara2, Cattaneo Antonino1, 2 *
1Scuola Normale Superiore, Pisa, Italy, 2European Brain Research Institute (EBRI) Rita Levi-Montalcini, Roma, Italy, 3Fondazione Santa Lucia IRCCS, Roma, Italy
*Corresponding author:
Antonino Cattaneo, Scuola Normale Superiore (SNS), Piazza dei Cavalieri 7, 56126 Pisa, Italy. Tel: +39 50 509320; Fax: +39 50 3153210
E-mail: antonino.cattaneo@sns.it
§ joint first authors

## Slide 2
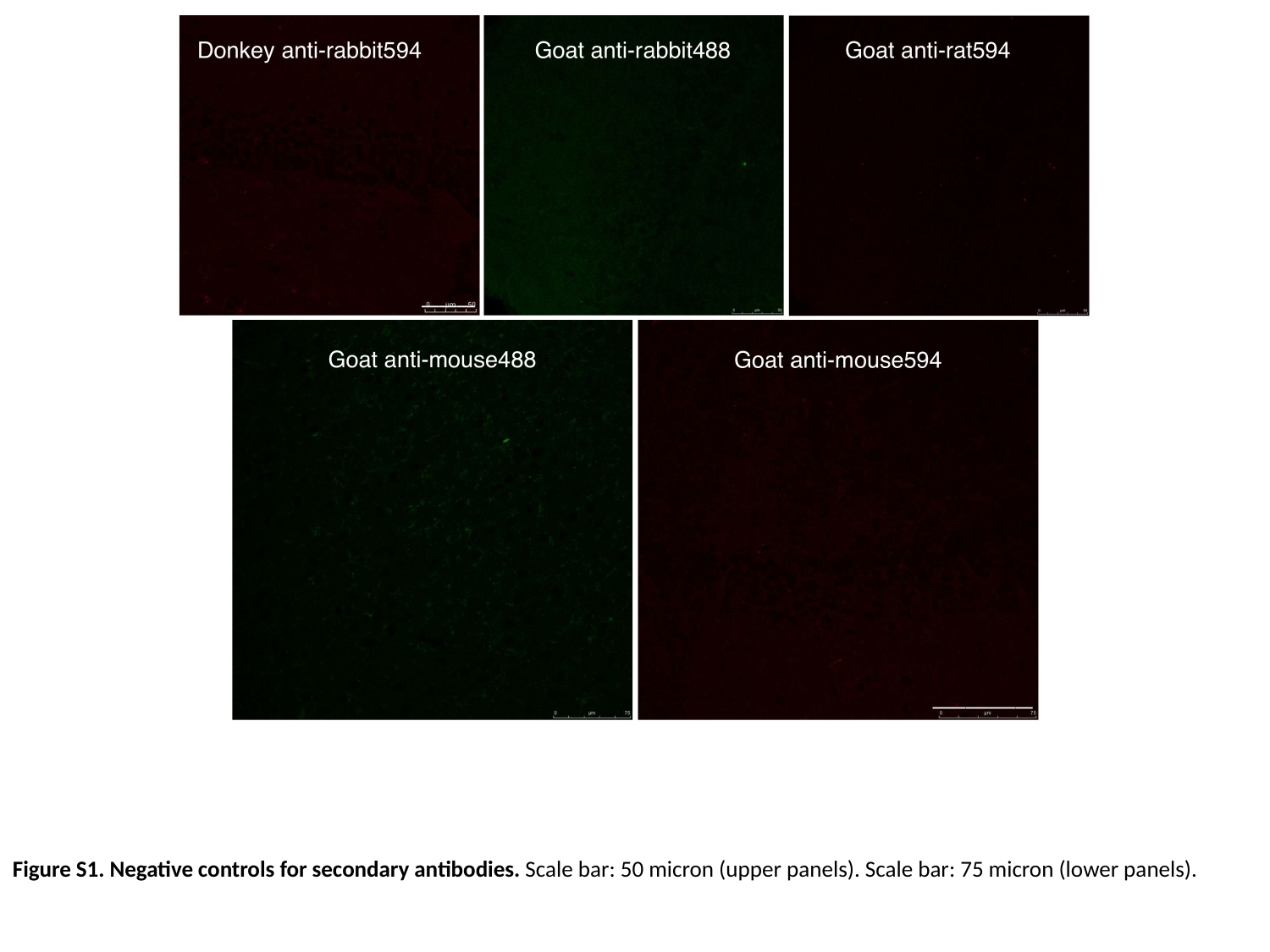

#
Figure S1. Negative controls for secondary antibodies. Scale bar: 50 micron (upper panels). Scale bar: 75 micron (lower panels).

## Slide 3
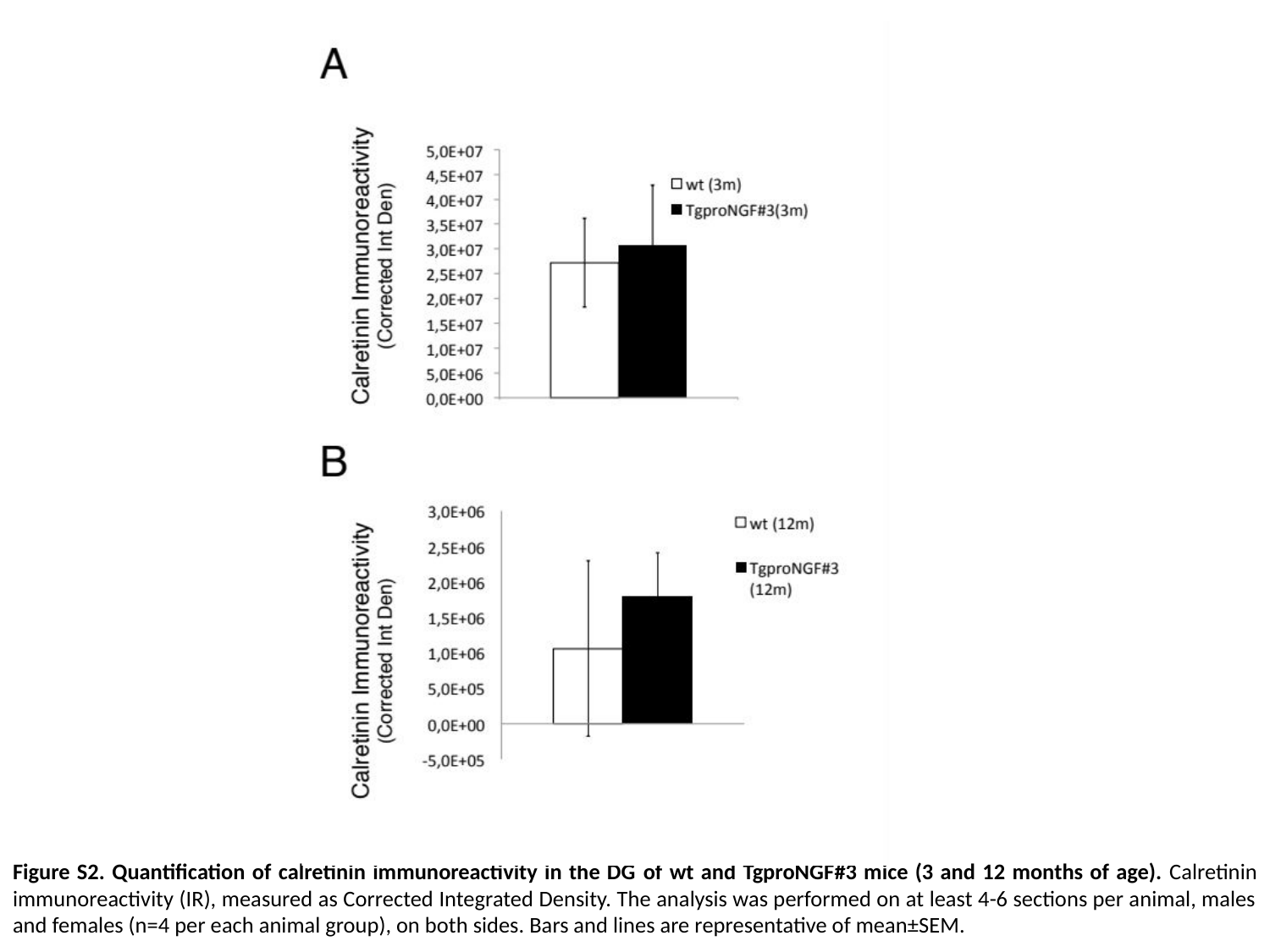

# Figure S2. Quantification of calretinin immunoreactivity in the DG of wt and TgproNGF#3 mice (3 and 12 months of age). Calretinin immunoreactivity (IR), measured as Corrected Integrated Density. The analysis was performed on at least 4-6 sections per animal, males and females (n=4 per each animal group), on both sides. Bars and lines are representative of mean±SEM.

## Slide 4
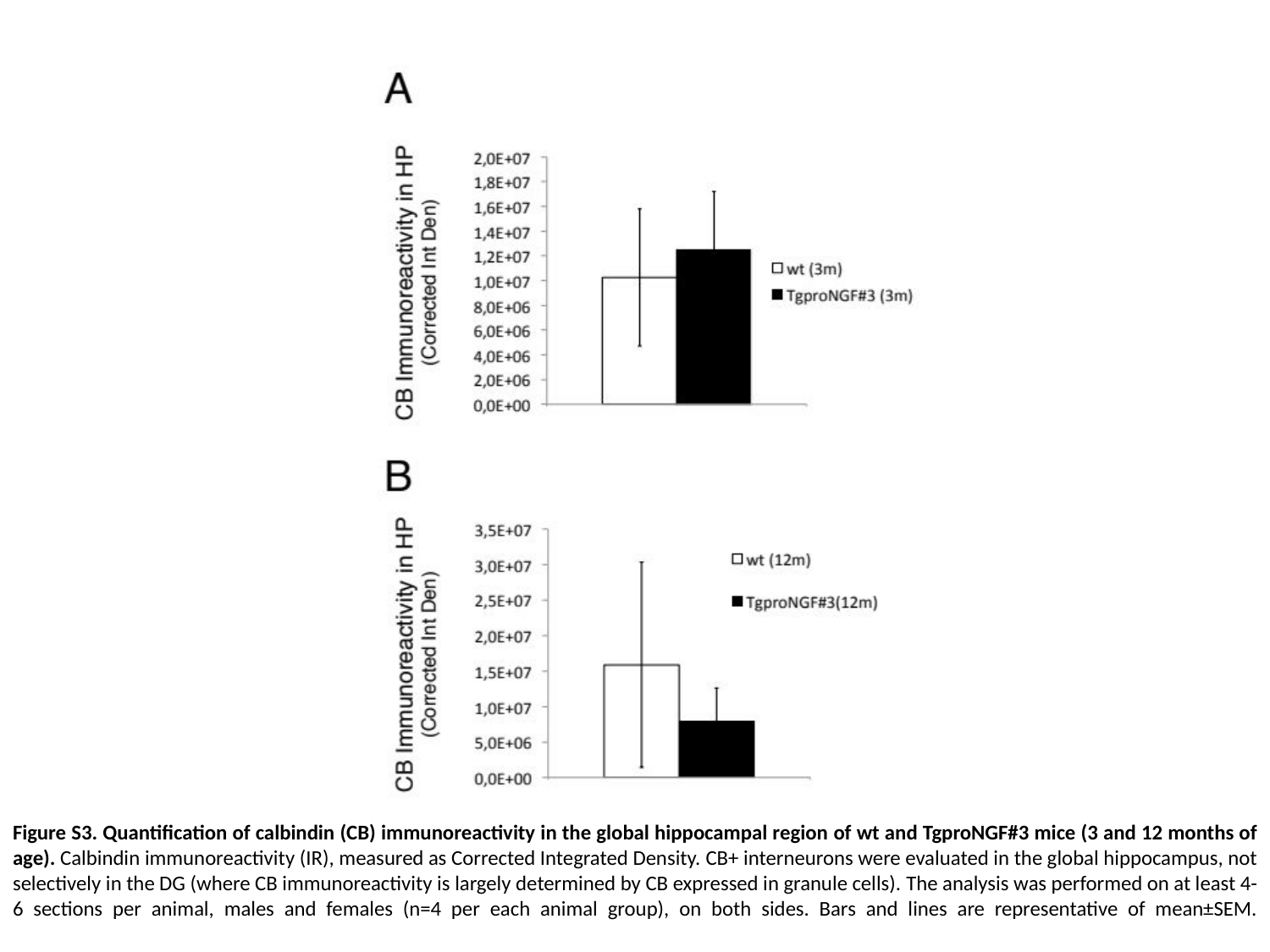

# Figure S3. Quantification of calbindin (CB) immunoreactivity in the global hippocampal region of wt and TgproNGF#3 mice (3 and 12 months of age). Calbindin immunoreactivity (IR), measured as Corrected Integrated Density. CB+ interneurons were evaluated in the global hippocampus, not selectively in the DG (where CB immunoreactivity is largely determined by CB expressed in granule cells). The analysis was performed on at least 4-6 sections per animal, males and females (n=4 per each animal group), on both sides. Bars and lines are representative of mean±SEM.

## Slide 5
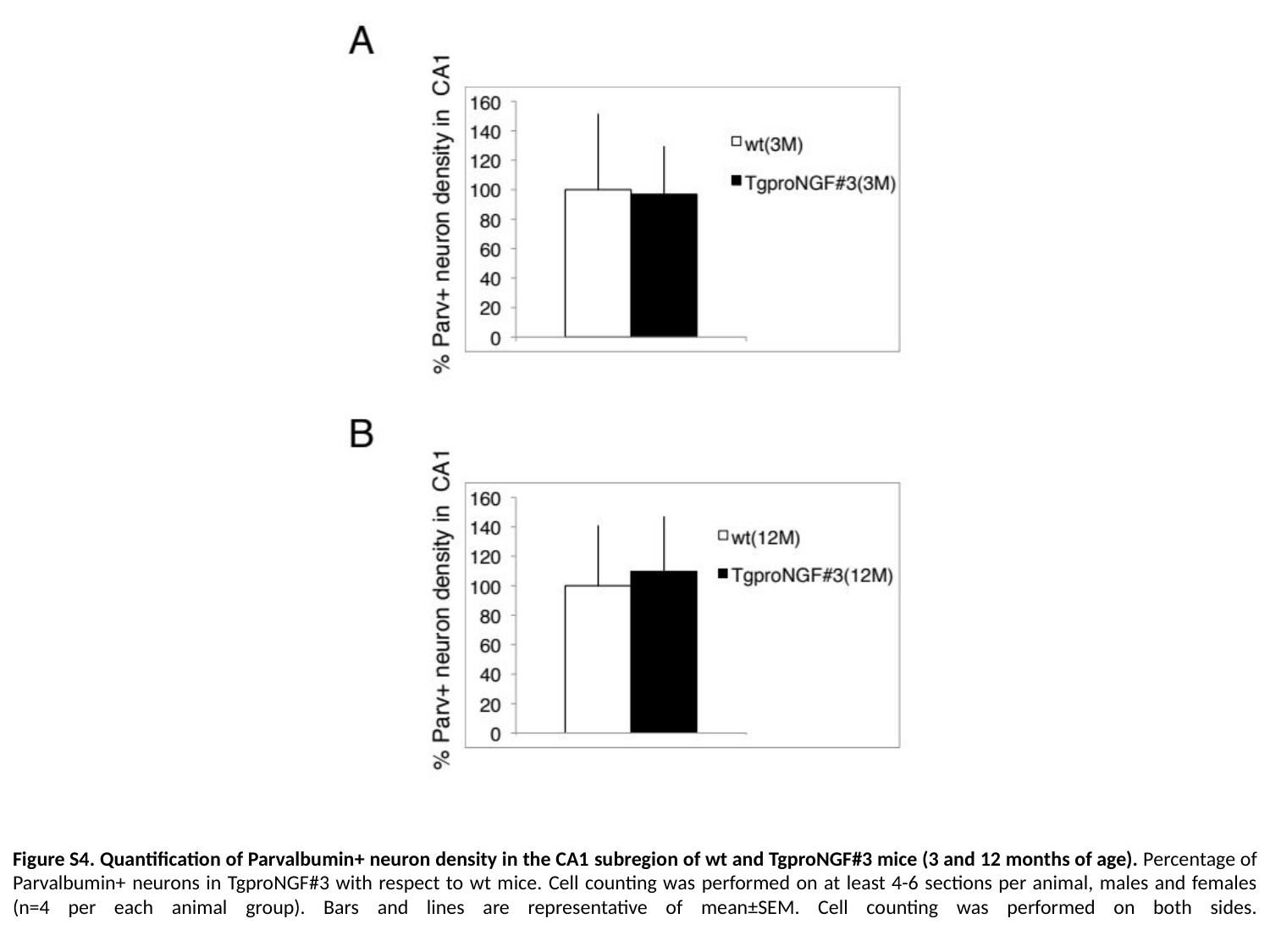

# Figure S4. Quantification of Parvalbumin+ neuron density in the CA1 subregion of wt and TgproNGF#3 mice (3 and 12 months of age). Percentage of Parvalbumin+ neurons in TgproNGF#3 with respect to wt mice. Cell counting was performed on at least 4-6 sections per animal, males and females (n=4 per each animal group). Bars and lines are representative of mean±SEM. Cell counting was performed on both sides.

## Slide 6
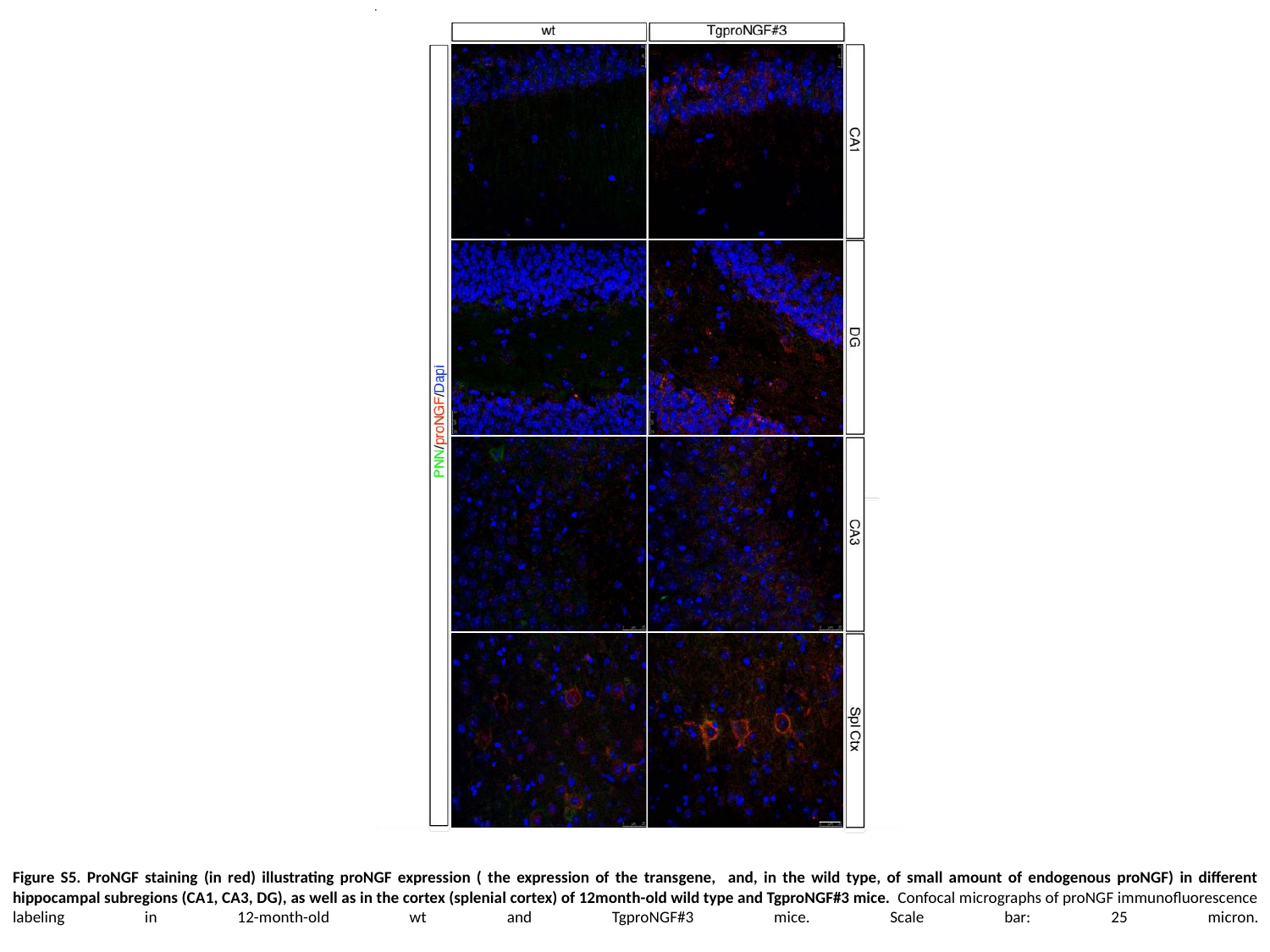

# Figure S5. ProNGF staining (in red) illustrating proNGF expression ( the expression of the transgene, and, in the wild type, of small amount of endogenous proNGF) in different hippocampal subregions (CA1, CA3, DG), as well as in the cortex (splenial cortex) of 12month-old wild type and TgproNGF#3 mice. Confocal micrographs of proNGF immunofluorescence labeling in 12-month-old wt and TgproNGF#3 mice. Scale bar: 25 micron.

## Slide 7
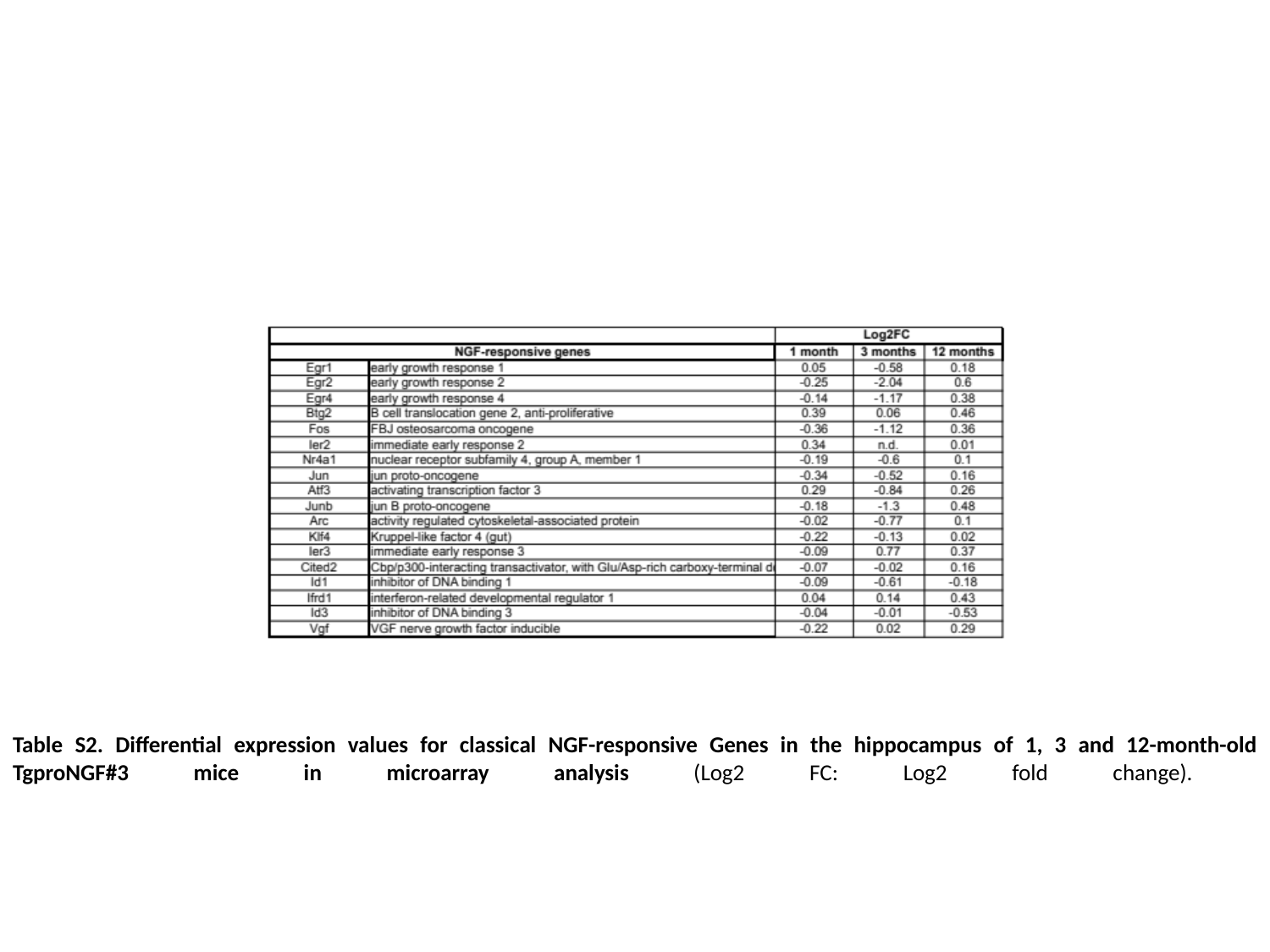

# Table S2. Differential expression values for classical NGF-responsive Genes in the hippocampus of 1, 3 and 12-month-old TgproNGF#3 mice in microarray analysis (Log2 FC: Log2 fold change).

## Slide 8
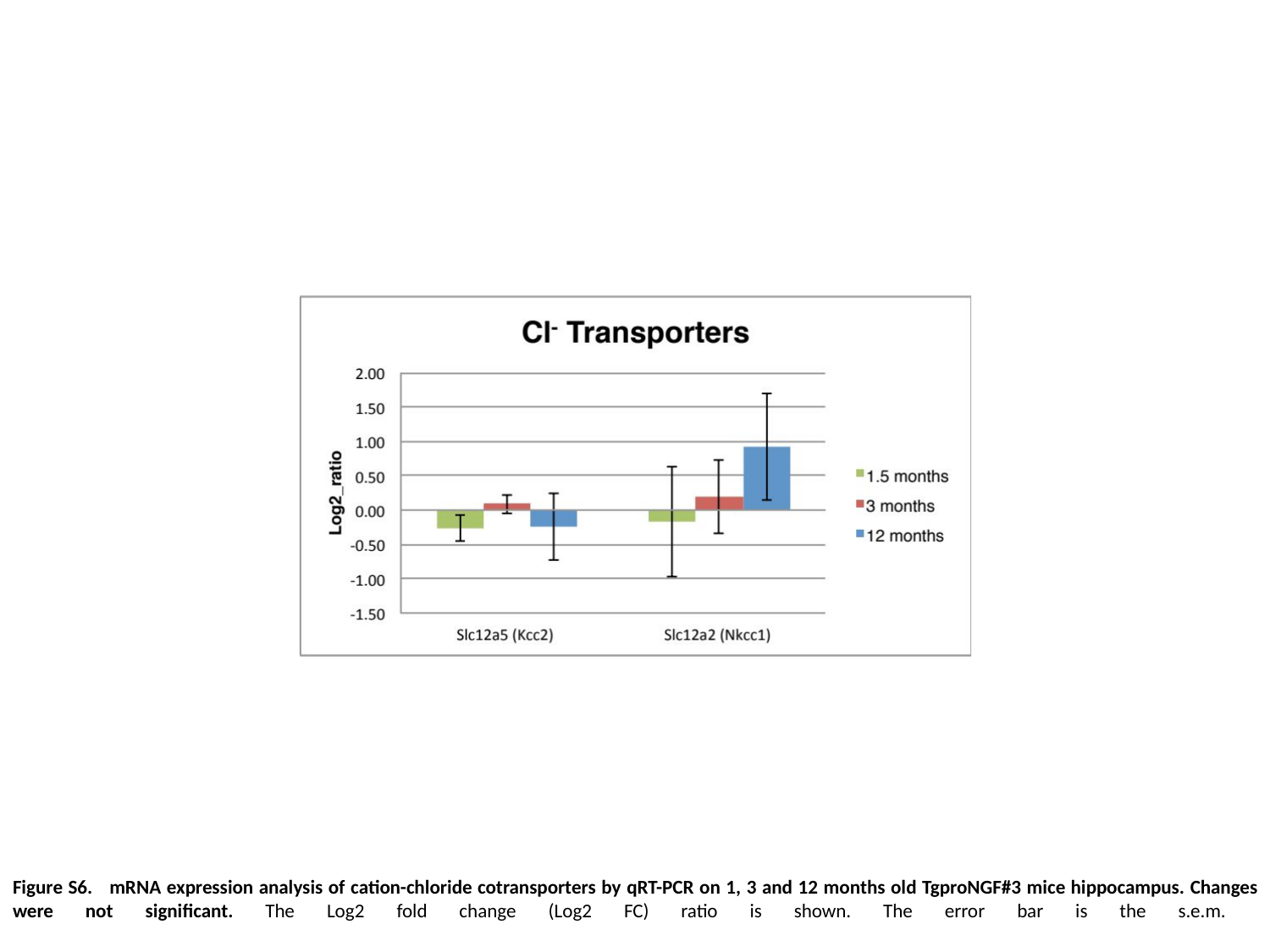

# Figure S6. mRNA expression analysis of cation-chloride cotransporters by qRT-PCR on 1, 3 and 12 months old TgproNGF#3 mice hippocampus. Changes were not significant. The Log2 fold change (Log2 FC) ratio is shown. The error bar is the s.e.m.

## Slide 9
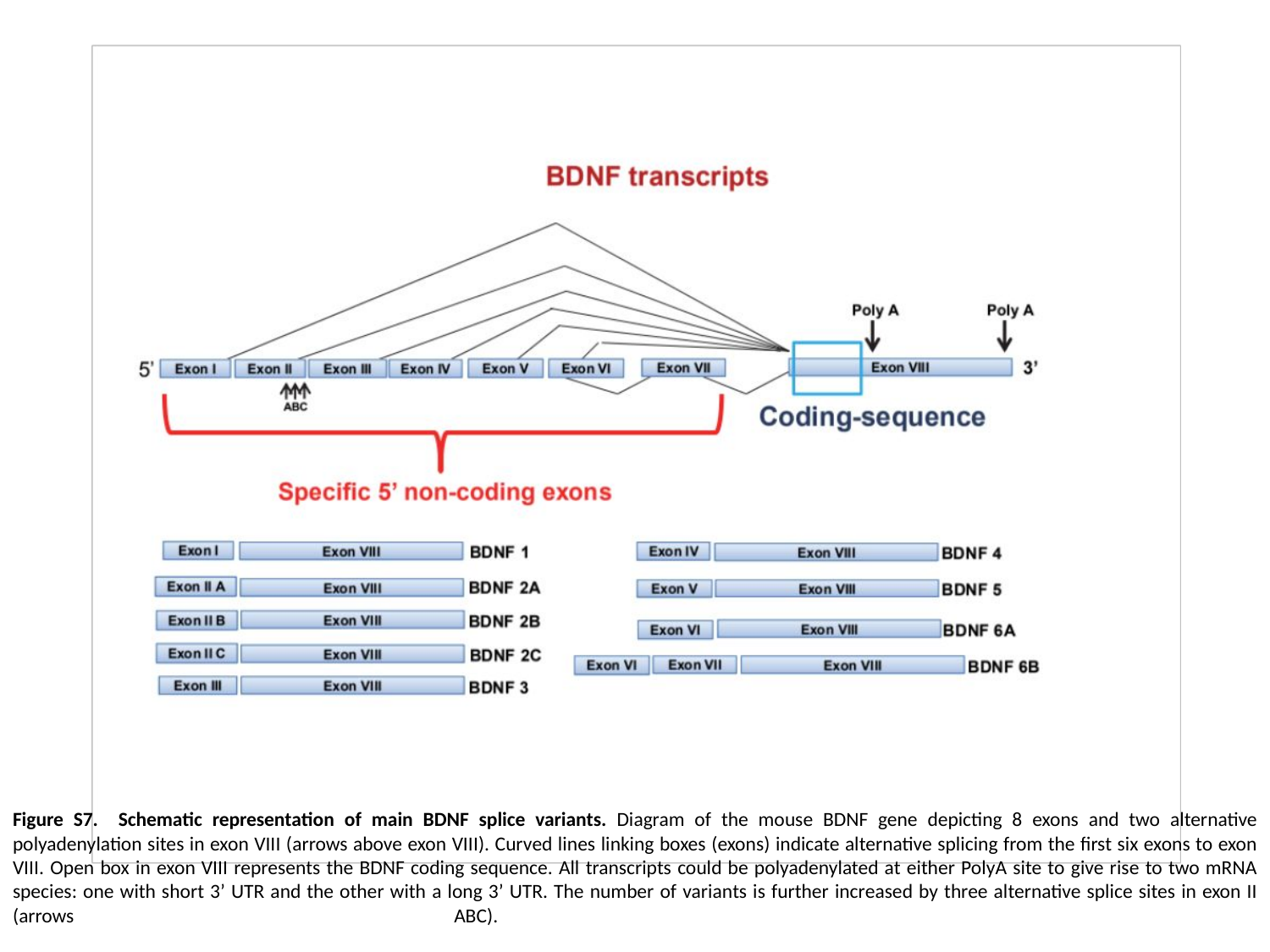

# Figure S7. Schematic representation of main BDNF splice variants. Diagram of the mouse BDNF gene depicting 8 exons and two alternative polyadenylation sites in exon VIII (arrows above exon VIII). Curved lines linking boxes (exons) indicate alternative splicing from the first six exons to exon VIII. Open box in exon VIII represents the BDNF coding sequence. All transcripts could be polyadenylated at either PolyA site to give rise to two mRNA species: one with short 3’ UTR and the other with a long 3’ UTR. The number of variants is further increased by three alternative splice sites in exon II (arrows ABC).

## Slide 10
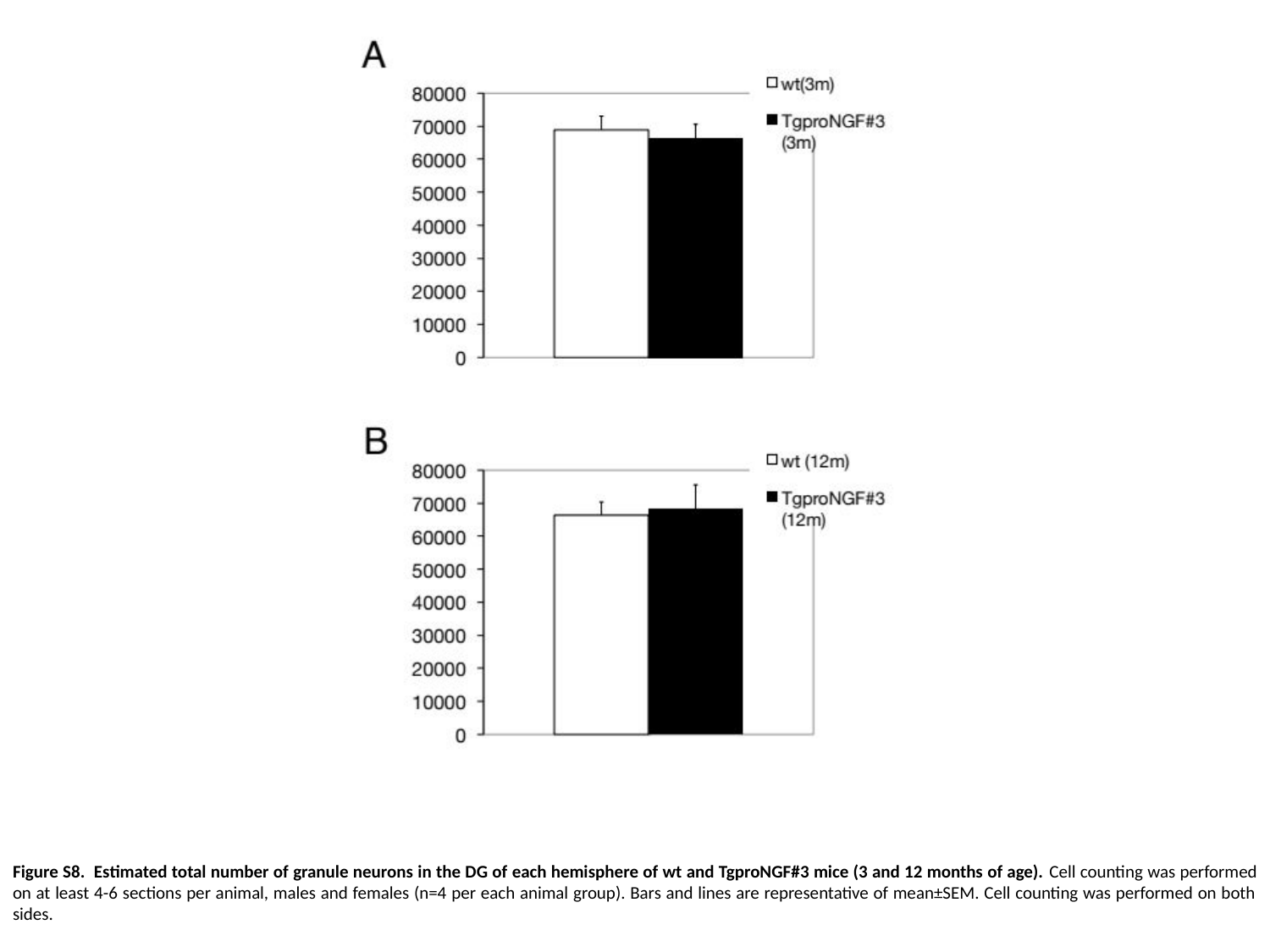

# Figure S8. Estimated total number of granule neurons in the DG of each hemisphere of wt and TgproNGF#3 mice (3 and 12 months of age). Cell counting was performed on at least 4-6 sections per animal, males and females (n=4 per each animal group). Bars and lines are representative of mean±SEM. Cell counting was performed on both sides.
